# Moderate regular physical exercise can help in alleviating the systemic impact of schistosomiasis infection on brain cognitive function

**DOI:** 10.3389/fimmu.2024.1453742

**Published:** 2025-01-31

**Authors:** Inssaf Berkiks, Nada Abdel Aziz, Blessing Moses, Tiroyaone Brombacher, Frank Brombacher

**Affiliations:** ^1^ Cytokines and Diseases Group, International Centre for Genetic Engineering and Biotechnology, Cape Town Component, Division of Immunology, Institute of Infectious Diseases and Molecular Medicine, Faculty of Health Sciences, University of Cape Town, Cape Town, South Africa; ^2^ Wellcome Centre for Infectious Diseases Research in Africa, Institute of Infectious Diseases and Molecular Medicine (IDM), Faculty of Health Sciences, University of Cape Town, Cape Town, South Africa; ^3^ Biotechnology Department, Faculty of Science, Cairo University, Cairo, Egypt

**Keywords:** shistosomiasis, morris water maze, neuroinfection, physical activities, *Schistosoma mansoni*

## Abstract

One of the major consequences of schistosomiasis is its impact on brain function, and despite its severity, the underlying mechanism(s) remain inadequately understood, highlighting a knowledge gap in the disease. The symptoms can vary from headaches to profound cognitive impairment. Besides, the potential influence of physical exercise in mitigating cognitive deficits has received little attention. In our study, we utilized a murine model of *Schistosoma mansoni* infection to investigate the cognitive impact of schistosomiasis. Our aims were multifaceted: to pinpoint the specific cognitive domains affected during the infection in adult mice, to unravel the complex interplay between glial and immune cells within the central nervous system (CNS), and crucially, to explore the potential therapeutic role of regular physical exercise in counteracting the deleterious effects of schistosomiasis on the CNS. Our findings unveiled that while acute infection did not disrupt simple and complex learning or spatial reference memory, it did induce significant deficits in recall memory—a critical aspect of cognitive function. Furthermore, our investigation unearthed profound alterations in the immune and glial cell populations within the CNS. Notably, we observed marked changes in CD4^+^ T cells and eosinophils in the meninges, as well as alterations in glial cell dynamics within the hippocampus and other brain regions. These alterations were characterized by heightened microglial activation, diminished astrocyte reactivity and a shift towards a proinflammatory milieu within the CNS. We also provided insights into the transformative potential of regular moderate physical exercise in partially alleviating cognitive and neuroinflammatory consequences of schistosomiasis. Remarkably, exercise decreased glial cell production of TNFα, suggesting a shift towards a less pro-inflammatory environment. Collectively, our study provided compelling evidence of the intricate interplay between schistosomiasis infection and cognitive function, underscoring the critical need for further exploration in this area. Furthermore, our findings demonstrated the positive effects of physical activities on mitigating the cognitive burden of schistosomiasis, offering new hope for patients afflicted by this debilitating disease.

## Introduction

Schistosomiasis is one of the most debilitating neglected tropical diseases, afflicting approximately 240 million individuals and resulting in around 280,000 deaths annually (1–3). Furthermore, it imposes a staggering burden of up to 4 million disability-adjusted life years (DALYs) (3). Schistosomiasis typically progresses from an acute phase characterized by non-specific symptoms like fever and muscle aches to a chronic phase where the worms reside in the host’s blood vessels, leading to organ damage (3). The pathology of schistosomiasis stems from the systemic immune response triggered by the presence of eggs trapped within the tissues leading to granuloma formation (4). Although it is rare, schistosomiasis can be also directly involved in the brain and spinal cord, causing a condition known as neuroschistosomiasis. Despite being under-diagnosed, this form affects at least 2-5% of the 200 million individuals infected worldwide (5, 6) making it the second most common presentation of *S. mansoni* infection (7).

The systemic effects of *S. mansoni* infection on neurological function can develop at any stage of the disease (5, 6). The key pathologic feature is driven by schistosome eggs in the central nervous system (CNS) resulting in granulomatous formation. Over time, these granulomas can cause focal neurological damage. Some neurological symptoms may also occur due to systemic inflammatory responses to the parasitic infection, which can be secondary to the release of cytokines and other inflammatory mediators (5, 6). There are two main forms of neuroschistosomias which are cerebral and spinal. Cerebral schistosomiasis is mediated by *S. japonicum*, and rarely *S. mansoni*, whereby the frontal and parietal lobes are often affected. Symptoms may include seizures, headaches, focal neurologic deficits, and altered mental status. On the other hand, spinal schistosomiasis is more commonly associated with *S. mansoni* infection whereby the thoracic spinal cord is predominantly affected. The clinical presentation can include paraparesis, quadriparesis, back pain, sensory deficits, and bladder and bowel dysfunction (5, 6). Of note, schistosomiasis also negatively impacts cognitive function. Research indicates that children who are moderately to heavily infected with helminths tend to score lower on cognitive function tests and achieve less in education compared to children who are either uninfected or lightly infected (8, 9). A recent attempt to understand the underlying mechanism demonstrated that *S. mansoni* infection led to impairment of spatial learning and memory capacity. The phenotype was associated with enhanced microglia and astrocytes number and reactivity (10). This was however characterized in mice at the post-natal stage, leaving the mechanisms underlying the impact of schistosomiasis on the adult brain unclear. It is worth mentioning that ​​murine models to study neuroschistosomiasis provides valuable insights due to their genetic tractability, allowing for the exploration of pathogen-host interactions and therapeutic strategies. However, one of the main limitations is the significant biological differences between murine and human hosts that may not fully recapitulate the complexity of human neurological involvement or the diverse clinical presentations seen in neuroschistosomiasis. Consequently, while murine studies can highlight potential mechanisms and treatments, they may always need translational validation directly on human patients.

Praziquantel is the mainstay drug for treating schistosomiasis. However, in cases of neuroschistosomiasis, its efficacy in reducing neurological symptoms is unclear, as the drug may not always effectively cross the blood-brain barrier (8). Corticosteroids are typically administered alongside praziquantel to reduce inflammation caused by the dying parasites (5, 6). However, corticosteroids carry significant side effects that can potentially harm the host, making the exploration of safe natural alternatives imperative. One such non-invasive approach known for its ability to downregulate immune response and confer protection against chronic inflammation is physical activities (PA) (11). Reports demonstrated that regular moderate exercise have protective and anti-inflammatory impacts, thereby enhancing CNS functionality (12). This is achieved through various pathways i) transient increase in IL-6 that drives the production of anti-inflammatory mediators IL-10 and Interleukin-1 receptor antagonist (IL-1RA), ii) stimulating the adrenal gland cortex and medulla to produce adrenaline and glucocorticoid, respectively, iii) reducing the expression of toll-like receptor on monocytes, and/or iv) diminishing the circulating number of pre-inflammatory monocytes. These pathways collectively contribute to the reduction of pro-inflammatory mediators and bolster the ability of immune cells within the CNS to adopt an anti-inflammatory phenotype (11, 12). Generation of the anti-inflammatory phenotype fosters neuroplasticity, neurogenesis, neuroprotection, and support hippocampus-mediated learning and memory (13, 14). The question of whether PA can effectively mitigate the pro-inflammatory impact of schistosomiasis on the brain remains open and warrants further investigation.

In the present study, we aimed to 1) dissect the impact of *S. mansoni* infection on the specific learning and memory domains in adult mice, 2) characterize the cellular changes in CNS in response to schistosomiasis infection, and to 3) finally to evaluate the impact of PA in alleviating schistosomiasis-induced changes in the CNS.

## Materials and methods

### Animals

Wildtype mice on BALB/c background were used, ad all mice were maintained in specific-pathogen-free barrier conditions in individually ventilated cages at the University of Cape Town biosafety level 2 animal facility. Experimental mice were sex and age-matched and used at 8 weeks of age. All the experimental work was done in strict accordance with the recommendations of the South African national guidelines and of the University of Cape Town practice for laboratory animal procedures as in ethics protocols, 020-007, approved by the Animal Research Ethics Committee of the Faculty of Health Science, University of Cape Town. All efforts were made to minimize animal suffering. Upon reaching the study experimental endpoint and/or the protocol-defined humane endpoint, animals were euthanized under this study by exposure to an excess of Halothane (4% in air) for 5 minutes. Death was confirmed either by neck dislocation or exsanguination by cardiac puncture. Death was not a pre-determined endpoint in any of the arms of this study.

### *S. mansoni* infection

Prior to percutaneous infection with *S. mansoni* cercariae, animals between 7 to 8 weeks age were anesthetized by intraperitoneal injection of a cocktail of Ketamine (100 mg/kg) and Xylazine (10 mg/kg) and monitored for 5 mins to confirm deep anesthesia. Anesthesia was confirmed by the absence of pedal reflex (toe pinch) and eyeblink reflex amid a regular respiratory rate. The anesthesia duration was of a maximum of 30 minutes. During the anesthesia phase, animals were exposed to an infra-red lamp to help them maintain their core body temperature. This procedure was performed and dully cared for by trained and authorized researchers. Then, mice were percutaneously infected via the abdomen, using stainless-steel rings, with 0 or 35 viable cercariae of a Puerto Rican strain of *S. mansoni* obtained from infected *Biomphalaria glabrata* snails (NMRI strain, NR-21962, provided by Biomedical Research Institute, Rockville, MD) for control or infected group, respectively. Post-infection, animals were monitored until regaining of consciousness and moistened food was added to the cage bedding.

### Morris Water Maze

The Morris Water Maze (MWM) is a widely-used behavioral testing procedure designed to study spatial learning and memory in rodents, particularly rats and mice (15). The MWM task involved a training phase where mice performed four swim trials 1 min each per day (with 5 minutes interval between trials) for 4 consecutive days to locate a plexiglass circular platform (10cm in diameter), which was placed approximately 0.5 cm below the water level in an open circular 123cm diameter MWM. The water temperature was controlled using an automated water heater and made to equilibrate with the room temperature maintained at 20-24˚C. During the training phase of the task, each mouse was allowed a maximum of 60 seconds to locate and climb onto the platform. Once the mouse had located the platform, it was given approximately 10 seconds to remain on the platform. Mice that failed to locate the platform within 60 seconds were gently guided to the platform and allowed to acclimatize for 10 seconds before returning to the home cage. During this phase, the test was measuring learning memory and spatial cues. On the 5th day, a probe trial was performed with the platform removed in order to test reference memory (observations were based on the number of and latency to platform crossings). Each mouse was given a maximum of 60 seconds in the MWM to find and cross the platform location. On days 6 and 7, the platform is placed in the quadrant opposite the original training quadrant, and the mouse was retrained for four trials each day (i.e. 60sec swim with approximately 5mins interval x 4 trials). On day 8 mice were introduced to the pool with a visible platform in a third quadrant, placed approximately 0.5 cm above water level to test learning memory. All data was recorded using the EthoVision XT 8 automated tracking system (Noldus Information Technology, VA).

### Experiments design

The MWM put mice to a swimming task for 8 consecutive days, hence right after day 8 of MWM we attribute potential biological effects to the immediate impact of physical activity. To evaluate the long-term effects of the initial physical activities on the animals, we repeated the MWM test at 11 weeks post-infection, during the chronic stage, to further examine any lasting impacts. Thus, our experimental design included physical activities as a factor, and infection status as another independent factor, generating 4 experimental groups: non-infected non-trained (NINT), non-infected trained (NIT), infected non-trained (INT), and infected trained (IT).

### Eggs detection

Mice were euthanized at acute stage (8 weeks post-infection). Eggs were purified from KOH digested liver, ileum, and spinal cord and counted at 40x magnifications as previously described (16–18). The method for determining collagen production through hydroxyproline content was conducted as outlined in reference (18). Briefly, liver samples of specific weight were hydrolyzed in 6 M hydrochloric acid at 110°C overnight, followed by filtration using Whatman filter papers. The resulting filtrate was then neutralized using 1% phenolphthalein and titrated with 10 M sodium hydroxide. A portion of this filtrate was combined with isopropanol and introduced to a solution of chloramine-T and citrate buffer (pH 6.0). To this mixture, Ehrlich’s reagent (comprising 25 g of p-dimethyl-amino-benzaldehyde and 37.5 ml of 60% perchloric acid) was added. The absorbance was measured at 570 nm employing a VersaMax microplate spectrophotometer from Molecular Devices. The concentration of hydroxyproline was determined using a standard of 4-hydroxy-L-proline (Calbiochem, San Diego, CA, US) with the results being reported in micrograms of hydroxyproline per weight of liver tissue, which contained 10^4 eggs.

### Physical exercise using Morris Water Maze

Cognitive function was assessed at the acute stage, 8 weeks post-infection, using the Morris Water Maze (MWM) task for 8 consecutive days to verify the immediate impact of exercise. To evaluate the long-term effects of the initial exercise on the animals, we repeated the Morris Water Maze (MWM) test at 11 weeks post-infection, during the chronic stage, to further examine any lasting impacts. using Morris Water Maze (MWM) platform for 8 consecutive days. MWM is a widely-used behavioral testing procedure designed to study spatial learning and memory in rodents, particularly rats and mice (15). The MWM task involved a training phase During the training phase, where mice were given performed four swim trials per day (with 5 minutes interval between trials) for 4 consecutive days to locate a plexiglass circular platform (10cm in diameter), which was placed approximately 0.5 cm below the water level in an open circular 123cm diameter MWM. The water temperature normally was controlled using an automated water heater and made to equilibrate with the room temperature maintained at 20-24˚C. During the training phase of the task, each mouse was allowed a maximum of 60 seconds to locate and climb onto the platform. Once the mouse has had located the platform, it was given approximately 10 seconds to remain on the platform. Mice that failed to locate the platform within 60 seconds were gently guided to the platform and allowed to acclimatize for 10 seconds before returning to the home cage. During this phase, the test help in measuring learning memory and spatial cues On the 5th day, a probe trial was performed with the platform removed in order to test reference memory (observations were based on helped in measuring learning memory and spatial cues. On the 5th day, a probe trial was performed with the platform removed in order to test reference memory (observations were based on the number of and latency to platform crossings). Each mouse was given a maximum of 60 seconds in the MWM to find and cross the platform location. On days 6 and 7, the platform is placed in the quadrant opposite the original training quadrant, and the mouse was retrained for four trials each day (i.e 60sec swim with approximately 5mins interval x 4 trials). On day 8 mice were introduced to the pool with a visible platform in a third quadrant, placed approximately 0.5 cm above water level to test learning memory. All data will be recorded using the EthoVision XT 8 automated tracking system (Noldus Information Technology, VA).

### Intestinal contractility

A measure of schistosomiasis infection was conducted 8 weeks post-infection. After euthanizing the mice, Approximately 1 cm of jejunum segments were removed from the small intestine of all groups. The smooth muscle contractile responses were measured using a water-jacketed organ bath (Panlab, Spain), connected to transducers and the PowerLabTM system (ADInstruments, Australia). This setup feeds and translates the signals to a computer for measuring tissue isometric tensions. The tissues were weighed on an analytical scale before being stimulated with varying concentrations of ACh (−9 to −3 LOG [M]) to determine the isometric contractile responses.

### Cells isolation

One day after MWM, animals were euthanized and perfused thoroughly with ice-cold PBS (pH 7.4) for 5 minutes. Following perfusion, heads were removed, and skulls were cleared of all tissue. Surgical scissors were utilized to sequentially remove the tops of the skulls in a clockwise manner. Subsequently, the skulls were promptly placed in ice-cold RPMI media. Meninges were meticulously extracted from the interior surfaces of the skulls and the brain surfaces using forceps (19). The hippocampus and prefrontal cortex were then separated from the brain parenchyma using surgical forceps. Part of hippocampus and prefrontal cortex was used for histology while the rest was used for single cell suspension. Single-cell suspensions from the meninges, hippocampus, and prefrontal cortex were prepared through enzymatic digestion in RPMI containing 220 U/mg Collagenase IV (Gibco, Waltham, Massachusetts), 13 U/mg DNase I (Sigma, St. Louis, Missouri), and 5% iFCS (inactivated fetal calf serum) (Gibco) in RPMI supplemented with 2mM MgCl2, 2mM CaCl2, 20% FBS, and 2 mM HEPES. The digested tissue was mechanically disrupted, filtered through a 100μm mesh, and then enriched for leukocytes by centrifugation (600 g, 10 minutes, no brakes) through 40% Percoll (Merck) (20).

### Flow cytometry

Antibodies used for flow cytometry analysis were as follows: CD3ϵ (500A2), CD4 (RM4-5), CD8α (53-6.7), CD19 (1D3), CD44 (IM7), CD62L (MEL-14), IFN-γ (XMG1.2), IL-4 (11B11), IL-13 (eBio13A), TNFα, CXCR5, CD11b, SiglecF, NK1.1, CD45, F4/80, MHC II, PD1, and GFAP purchased from BD Biosciences (Franklin Lakes, New Jersey) and eBioscience (San Diego, California). For staining of cell surface markers, cells (1 x 10^6^) were labeled and washed in PBS containing 1% BSA (Roche, Switzerland) and 0.1% NAN_3_ (FACS buffer). For detection of intracellular cytokines, cells were seeded at a density of 2 x 10^6^ cells/well in a complete RPMI culture medium and stimulated with 50 ng/ml phorbol myristate acetate (PMA), 250 ng/ml ionomycin and 200 µM monensin (all from Sigma) for 8-12 hr at 37°C in a humidified atmosphere containing 5% CO_2_. After the incubation period, cells were harvested, washed, fixed in 2% (w/v) paraformaldehyde, permeabilized with 0.5% saponin buffer, and then stained for cytokine production as previously described (17, 18). The acquisition was performed using BD LSRFortessa (BD Biosciences), and data were analyzed using FlowJo software (Treestar, Ashland, Oregon). Uniform Manifold Approximation and Projection (UMAP) was used for data visualization. It is a nonlinear dimensionality-reduction technique (21) available as a FlowJo plugin.

### Quantitative real-time RT-PCR

RNA from parenchyma single cell suspension was reverse transcribed by Transcriptor First Strand cDNA Synthesis Kit (Roche) according to manufacturer’s instructions. Real-time reverse transcribed PCR (QRT-PCR) was performed with LightCycler 480 SYBR Green I Master mix in LightCycler 480 II (Roche) and gene-specific primers (IDT, CA, USA). Fold change in gene expression was calculated by the ΔΔCt method and normalized to *Hprt1* which was used as internal control as described (22).

The primers used are as follow (**Table 1**):

**Table 1:** The primers used in qPCR.

No	Gene	Direction	Sequences
1	HPRT	Forward	5’– GTT GGA TAT GCC CTT GAC – 3’
		Reverse	5’– AGG ACT AGA ACA CCT GCT – 3’
2	Muscarinic M5	Forward	5’ – CTC TGC TGG CAG TAC TTG GTC – 3’
		Reverse	5’ – GTG AGC CGG TTT TCT CTT CTT – 3’
3	Muscarinic M2	Forward	5’ – TGA AAA CAC GGT TTC CAC TTC – 3’
		Reverse	5’ – GAT GGA GGA GGC TTC TTT TTG – 3’
4	Muscarinic M 1	Forward	5’ – GGA CAA CAA CAC CAG AGG AGA – 3’
		Reverse	5’ – CGA GGT CAC TTT AGG GTA GGG – 3’
5	IL-4	Forward	5’ –TCG GCA TTT TGA ACG AGG TC– 3’
		Reverse	5’ – GAA AAG CCC GAA AGA GTC TC– 3’
6	IL-6	Forward	5’ – CGT GGA AAT GAG AAA AGA GTT GTG– 3’
		Reverse	5’ – ATC TCT CTG AAG GAC TCT GGC T – 3’
7	IL-13	Forward	5’ – CTC CCT CTG ACC CTT AAG GAG – 3’
		Reverse	5’ – GAA GGG GCC GTG GCG AAA CAG – 3’
8	CCL2	Forward	5’ – CTC TCT CTT CCT CCA CCA CCA T – 3’
		Reverse	5’ – TGG GGC GTT AAC TGC ATC TG – 3’
9	TNFα	Forward	5’ – TCT CAT CAG TTC TAT GGC CC – 3’
		Reverse	5’ – GGG AGT AGA CAA GGT ACA AC– 3’

### Tissue homogenate for cytokine analysis

Brain parenchyma (hippocampus and prefrontal cortex) was collected and homogenized in RIPA buffer. Cytokines (TNFα, IL-6, and MCP all from BD Pharmingen) were measured in the protein extracts by sandwich ELISA as described previously (17, 18). Cytokine values were normalized according to the protein content measured by Pierce BCA Protein Assay Kit (Thermo Fisher Scientific, catalogue no. 23225).

### Immunofluorescence

First, mice were anesthetized using isoflurane and perfused with ice-cold PBS and 4% paraformaldehyde (PFA) through the heart. Next, free-floating coronal sections from the brain or spinal cord were sectioned at 30 µm using a Leica cryostat. The sections were then immersed in a blocking solution consisting of PBS with 2% Normal Goat Serum, 1% BSA, 1% Triton-X, 0.05% Tween-20, and 0.05% sodium azide for 1 hour at room temperature. The sections were subsequently incubated overnight at 4°C with primary antibodies, including rabbit anti-Ach (Abcam), rabbit anti-Iba1(Abcam) at a concentration of 1:500 and 1:400, and rabbit anti-Iba1(Abcam), at a concentration of 1:500 and 1:400, respectively. After washing the sections with PBS, they were incubated with fluorophore-conjugated secondary antibodies at a dilution of 1:400 for 2 hours. Finally, the sections were stained with DAPI at a concentration of 10 μg/ml for 10 minutes at room temperature, mounted on glass slides using moviol and antifade mounting medium from Thermo Fisher Scientific.

### Immunohistochemistry

Brain or spinal cord tissue was sectioned at a thickness of 9 µm using OCT compound and a cryostat. The sections were mounted on glass slides and dried for 2 hours. Following this, the slides were stained in 0.1% cresyl violet solution for 5 minutes, then rinsed with running distilled water. The sections were further dehydrated using graded alcohols, cleared in xylene, and finally mounted with antifade medium (Mowiol).

### Statistics

Statistical analysis was conducted using GraphPad Prism 6 software and SPSS 20. Data were calculated as mean ± SEM. Statistical significance was determined using the unpaired Student’s t-test and One-Way ANOVA with Bonferroni’s post-test after testing for normality and homogeneity in one category independent data. Two-way ANOVA was used to compare two categorical independent variables (infection and PA), and also the interaction between them, defining differences to uninfected mice as significant (*, *P* ≤ 0.05; **, *P* ≤ 0.01; ***, *P* ≤ 0.001). Two-way ANOVA was also employed to examine the interaction effects of infection and training, enabling an understanding of both the separate and combined effects of these factors. This comprehensive approach provided a nuanced understanding of the collective impact of infection and training on experimental outcomes.

## Results

### Acute schistosomiasis causes recall memory impairment

In order to understand how systemic Schistosomiasis infection may affect the brain function, especially the behavior, we conducted MWM test at the acute stage (8 weeks post-infection to assess cognitive abilities.

**Figure 1:**
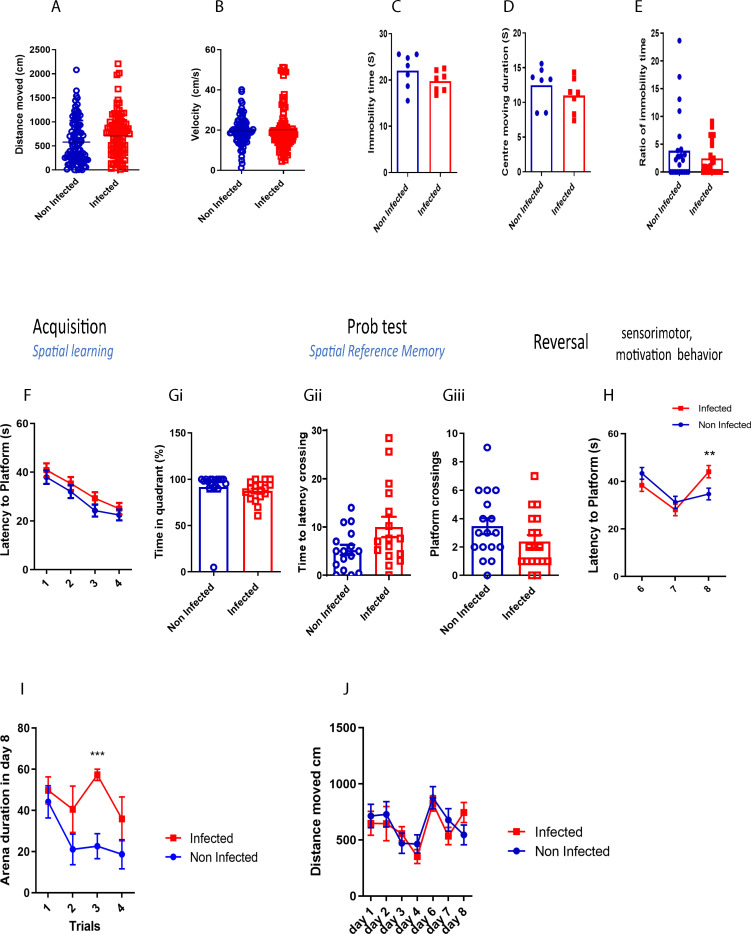
Acute schistosomiasis infection can impair recall memory. **(A)** Distance moved by the mice in MWM, **(B)** the villosity, **(C)** Immubility time, **(D)** Centre moving duration, **(E)** Ratio of immobility time, **(F)** Spatial learning was assessed during the first four days whereby latency to platform was assessed. A probe test was then done on day 5, and **(Gi)** the time percentage spent in the right training quadrant, **(Gii)** the latency to cross the virtual platform, and **(Gii)** the number of times the mice crossed the platform were recorded. **(H)** Reversal trial was then performed from day 6 to day 8 and the latency to the new platform was measured. **(I)** Duration spent on the arena during each trial on day 8. **(J)** Distance travelled in each day. Results are pooled from two different experiments with 6-8 mice per group. Data are expressed as mean ± S.E.M. NS, P > 0.05; *P < 0.05, ** *P* < 0.001, ***P < 0.0001 by two-tailed unpaired *Student t* test and repeated measures ANOVA.

The MWM analysis revealed some key findings. Firstly, *S. mansoni* infection did not significantly alter locomotor activity, as shown by the consistent distance moved (**Figure 1A**). Similarly, velocity measurements (**Figure 1B**) indicated no signs of anxiety-like behavior. Furthermore, there were no significant differences in immobility time (**Figure 1C**), center-moving duration (**Figure 1D**), or immobility time ratio (**Figure 1E**), suggesting the absence of any phenotypes-like behavior in infected mice.

Both infected and non-infected mice demonstrated comparable spatial learning abilities, with a similar reduction in the time taken to reach the platform across learning sessions (**Figure 1F**). To further assess spatial reference memory, a probe test was performed 24 hours after the last learning trial. No significant differences were observed between the groups in time spent in the platform’s quadrant (**Figure 1Gi**), latency to cross the platform area (**Figure 1Gii**), or the number of platform crossings (**Figure 1Giii**), indicating that spatial memory remained intact in infected mice after 8 weeks post infection.

However, during the reversal trial, when the platform was relocated to a different quadrant, infected mice displayed a significant increase in latency to find the platform on day 8 compared to controls (**Figure 1H**). Additionally, infected mice spent more time in the arena (**Figure 1I**), although the distance they travelled remained similar to that of non-infected mice (**Figure 1J**). These results suggest that while complex learning abilities were not impaired, schistosomiasis infection negatively impacted recall memory.

To our knowledge, this is the first study to dissect cognitive behavior in this context and shed light on the impact of schistosomiasis on recall system rather than the entire cognitive outcomes.

### Impact of physical activities on peripheral and CNS responses following schistosomiasis infection

Research shows that schistosomiasis infection harms intestinal function by causing inflammation and fibrosis, which disrupt normal muscle contractions needed for gut movement. When parasite eggs lodge in intestinal tissues, they trigger immune responses that lead to chronic inflammation, tissue damage, and changes in muscle activity, impairing digestion and nutrient absorption. In our study, we specifically looked at how physical activity (PA) impacts intestinal contractility after infection. Surprisingly, despite infection, the hypercontractility of the intestine was similar between non-infected, non-trained (NINT) and infected, non-trained (INT) groups (**Supplementary Figure S1A**). These results suggest that at this stage of infection, the presence of parasite eggs may not significantly alter muscle contractility. However, as noted in other studies, exercise slightly increased hypercontractility in both non-infected trained (NIT) and infected trained (IT) groups (**Supplementary Figure S1A**). While increased intestinal contractility can enhance gut motility and digestion, the implications of this change in the context of infection warrant further investigation.

Considering the potential link between enhanced intestine contractility and the expulsion of schistosomiasis eggs (23), we examined the effects of PA on egg burden and egg-driven immunopathology. Unexpectedly, our findings indicated that PA led to an increase in egg burden in the liver (**Supplementary Figure S1B**) and small intestine (**Supplementary Figure S1C**) in the IT group compared to the INT group. Furthermore, histopathological analysis revealed that training exacerbated liver granuloma size (**Supplementary Figures S1F, G**) and increased tissue fibrosis in the liver (**Supplementary Figure S1H**). These unexpected outcomes underscore the intricate relationship between PA and schistosomiasis progression, necessitating further exploration to unravel underlying mechanisms and potential implications for disease management.

**Figure 2:**
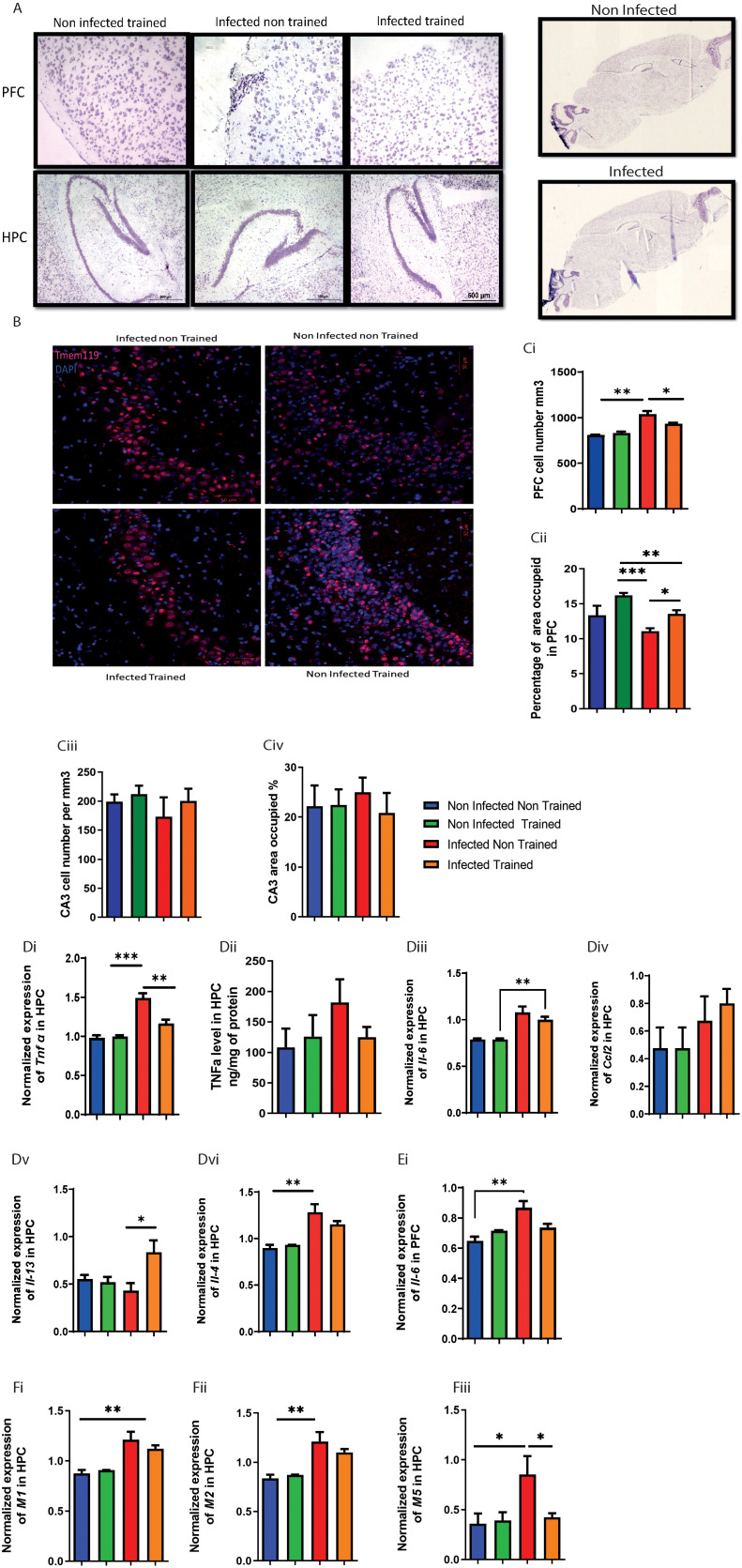
Impact of schistosomiasis infection and/or physical activities on brain parenchyma. **(A)** Eggs infiltration in prefrontal cortex (upper row) and hippocampus (lower row) was assessed using H&E staining (original magnification 500um). **(B)** Immunofluorescence staining of CA3 area in hippocampus to to show Tmem 119 positive cells area occupied 50um, (the quantification of cell presence within a defined spatial with 50um dept, specifically calculated as the number of cells per cubic millimeter (mm³) **(Ci)** Total cell number in mm^2^ and **(Cii)** area occupied in prefrontal cortex. **(Ciii)** Total cell number in mm^2^ and **(Civ)** area occupied in hippocampus. **(Di)** *Tnfa* mRNA expression relative to HPRT housekeeping gene determined using qRT-PCR from hippocampus. **(Dii)** TNFα concentration in hippocampus homogenate using ELISA. mRNA expression level of **(Diii)** *Il6*, **(Div)** *Ccl2*, **(Dv)** *Il13*, and **(Dvi)** *Il4* relative to HPRT housekeeping gene determined using qRT-PCR in hippocampus. mRNA expression level of **(Ei)** *Il6* relative to HPRT housekeeping gene determined using qRT-PCR in prefrontal cortex. Muscarinic receptors expression was assessed using qPCR, mRNA expression level of **(Fi)** *M1*, **(Fii)** *M2*, and **(Fiii)** *M5* relative to HPRT housekeeping gene determined using qRT-PCR in hippocampus. Results are pooled from two different experiments with 6-8 mice per group. Data are expressed as mean ± S.E.M. NS, P > 0.05; *P < 0.05, **P < 0.001, ***P < 0.0001 by two-way ANOVA followed by Bonferroni.

In our investigation of egg infiltration in the central nervous system (CNS), specifically the spinal cord and brain parenchyma, histological assessments yielded noteworthy results. As we expected, there was a lack of egg infiltration in the spinal cord (**Supplementary Figures S2A, B**) and brain parenchyma, particularly in the prefrontal cortex (PFC) and hippocampus (HPC), for both the infected non-trained (INT) and infected trained (IT) groups (**Figure 2A**).

Our study found that while schistosomiasis infection and PA did not affect intestinal muscle contractility in non-trained infected groups. Exercise increased egg burden in the liver and intestines, worsened liver granuloma size, and increased fibrosis, highlighting a complex interaction between PA and infection progression.

### Impact of schistosomiasis infection and physical activity on cholinergic system and cytokine expression in the nervous system

We next analyzed the impact of schistosomiasis infection and its interaction with PA on the cholinergic system. In spinal cord, the number of cells producing acetylcholine (ACh) showed a significant increase in the INT group compared to the NINT group (**Supplementary Figures S2Ci-C).** This indicated that schistosomiasis infection affected ACh production in the spinal cord. However, PA in the IT group restored ACh production to the baseline level, suggesting that exercise could mitigate the impact of infection on ACh production (**Supplementary Figures S2C-Ci**). On other hand, the expression of muscarinic receptors (*M1*, *M2*, and *M5*) (**Figures 2Fi-Fiii**) was examined in the HPC and PFC (**Supplementary Figures S2Diii, Dvi**). In the hippocampus, M1, M2, and M5 expression levels were remarkably increased in the INT group compared to the NINT group. However, M5 was the only receptor to decrease significantly in the IT group with PA, and statistical analysis showed a significant interaction effect. This suggests that while M1 and M2 are generally upregulated with infection, M5 expression may be uniquely modulated by the combination of infection and PA. For PFC, no change was noted in either *Ccl2*, *Il13*, *Il4*, *M1*, *M2*, *or M5* expression between the different groups **Supplementary Figures S2Di, Dvi**.

We further characterized cytokine expression in brain parenchyma. Significant effect was observed in the expression of TNFα in the HPC, with PA modulating its impact on *tnfα* expression dependent on infection presence (**Figure 2Di**). PA restored *tnfα* expression to baseline levels compared to the INT group (**Figure 2Di**). Similar, but not significant, *tnfα* trend was noted between the different groups (**Figure 2Dii**).

For *Il6*, the INT group exhibited a significant elevation in *Il6* expression compared to the NINT group in HPC (**Figure 2Diii**) and PFC (**Figure 2Ei**). However, PA did not significantly alter *Il6* expression. Training of the infected mice helped in partially restoring *Il6* expression (**Figures 2Diii, Ei**). Similar to *Il6*, the INT group showed a significant increase in *Il4* expression compared to the NINT group (**Figure 2Dvi**), and PA did not have a significant impact on *Il4* expression.

Analyzing the expression of *Ccl2* (**Figure 2Div**) and *Il13* (**Figure 2Dv**), no significant differences were observed between the INT, NINT, and NIT groups. However, it was worth noting that PA in the infected group significantly enhanced *Il13* expression compared to the rest (**Figure 2Dv**). In summary, Muscarinic receptors, particularly the M2 subtype, can modulate inflammation by inhibiting pro-inflammatory cytokines like TNFα and IL-6. Conversely, elevated cytokines can alter muscarinic receptor expression and function, impacting neuroinflammation and cognitive processes. This reciprocal relationship suggests that schistosomiasis-induced cytokine changes may influence muscarinic receptor activity, affecting neuroinflammatory responses and brain function.

### Physical activities helps reduce the impact of schistosomiasis on microglia and myeloid cell phenotypes in the brain

In the prefrontal cortex, PA’s impact on cell numbers depended on the presence of infection (**Figures 2B, 2Ci**), with infection and PA independently influencing microglia accumulation (**Figures 2B, 2Ci**). Moreover, the INT group exhibited higher cell numbers compared to the non-infected trained (NIT) and non-infected non-trained (NINT) groups (**Figures 2B, 2Ci**).

**Figure 3:**
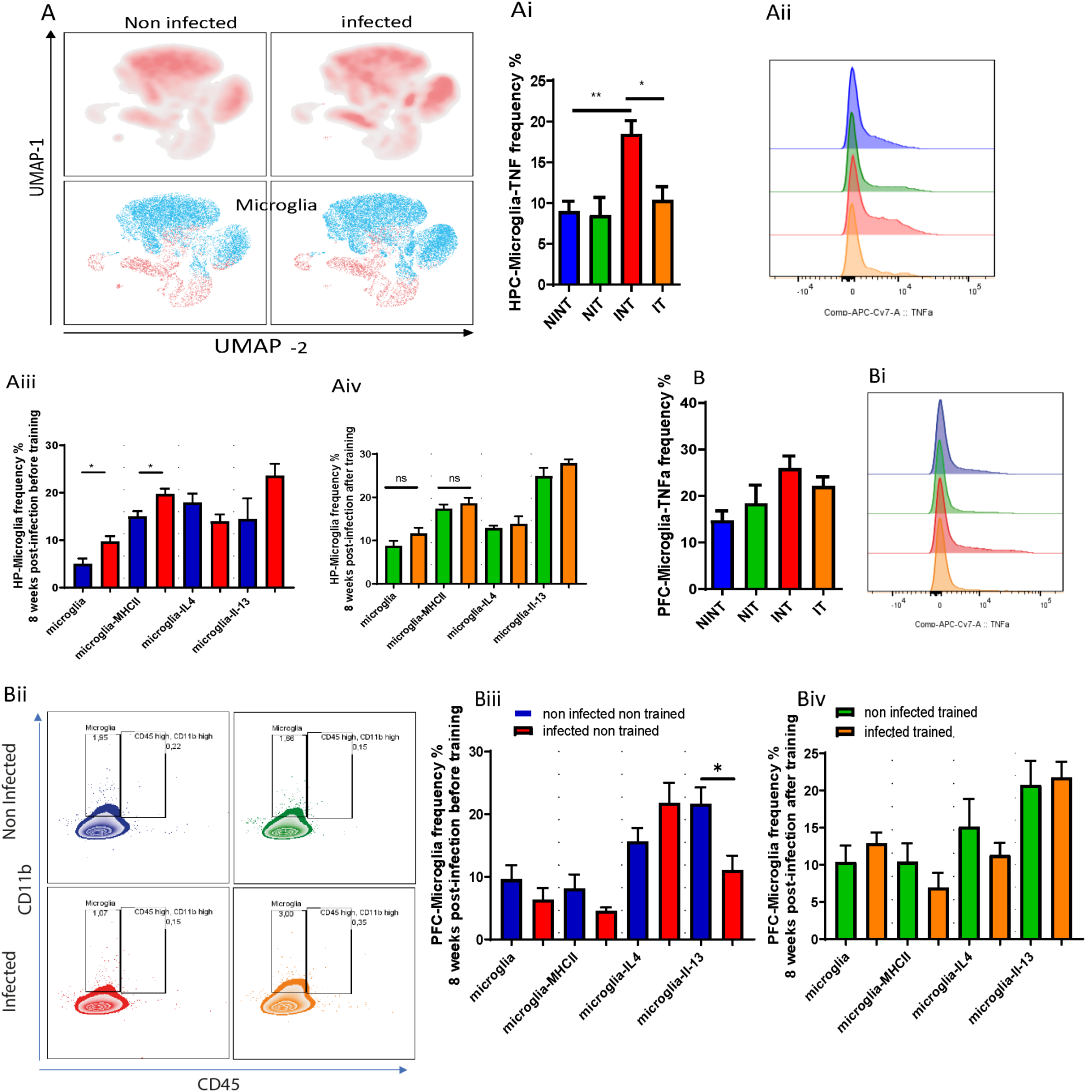
The impact of schistosomiasis infection and/or physical activities on microglia and myeloid cells phenotype. Wildtype BALB/c mice were infected with 0 or 35 *S. mansoni* cercariae and then euthanized 8 weeks post-infection and different brain regions were collected for characterization using flow cytometry. **(A)** UMAP visulaization of Microglia in hippocampus (HPC) using flow cytometry. **(Ai)** Frequency of microglia producing TNFα in hippocampus. **(Aii)** TNFα expression by microglia depicted in **(Aii)**. **(Aiii)** microglia frequency, MHC class II expression, IL-4, and IL-13 production before and after PA in hippocampus **(Aiv)**. Frequency of microglia producing TNFα in prefrontal cortex **(B)**. TNFα expression by microglia depicted in **(Bi, Bii)** Representative flow cytometry of microglia and CD45^+^ CD11b^+^ population. **(Biii)** microglia frequency, MHC class II expression, IL-4, and IL-3 production before and after PA in prefrontal cortex **(Biii, Biv)**. Results are pooled from two different experiments with 6-8 mice per group. Data are expressed as mean ± S.E.M. NS, P > 0.05; *P < 0.05, **P < 0.001 by two-way ANOVA followed by Bonferroni or non-parametric test after testing the homogeneity and normality.

The occupied area or cell distribution in the PFC revealed significant interaction effects, indicating the dependency of PAs impact on infection presence (**Figure 2Cii**). Additionally, main effects of PA and infection were observed, influencing the occupied area independently (**Figure 2Cii**). The INT group had a reduced occupied area compared to the NIT and IT groups (**Figure 2Cii**). Microglia in the HPC were not significantly affected by infection or PA (**Figures 2Ciii, Civ**), indicating the resilience of this cell population to the experimental conditions. Next, we delved into the impact of schistosomiasis infection in brain cells parenchyma using flow cytometry (**Figures 3A, Bii**). The findings revealed a significant increase in microglia frequency (**Figure 3Aiii**), TNFα production (**Figures 3Ai, Aii**), and activation status, marked by higher MHC class II (MHCII) expression (**Figure 3Aiii**) in HPC in response to schistosomiasis infection compared to the NINT group. In contrast, PA had no effect on microglia activation level in infected animals (**Figure 3Aiv**).Notably, a statistical analysis highlighted a significant interaction effect between the infection and training for TNFα production in the HPC (**Figures 3Ai, Aii**),. Of interest, and consistent with our previous observation (**Figure 2Di**), PA in IT group restored TNFα production to a level similar to NINT and NIT groups. Furthermore, PA did not impact microglia MHCII expression or the production of IL-4 or IL-13 in the HPC (**Figures 3iii, Aiv**). In the PFC comparable microglia frequency (**Figures 3Bii, Biii**), activation (**Figure 3Biv**), and TNFα production (**Figures 3B, Bi**) were observed in response to schistosomiasis infection and/or PA. Statistical analysis did not reveal significant effects on TNFα production in the PFC (**Figure 3Bi**).

Considering the anticipated type 2 immune response induced by *S. mansoni* infection, we examined microglia’s production of IL-4 and IL-13. Surprisingly, infected non-trained animals produced similar levels of IL-4 and IL-13 in the HPC compared with the non-infected group (**Figure 3Aiii**). In the PFC, while no significant change in IL-4 production was noted, the INT group exhibited a notable reduction in IL-13 production (**Figure 3Biii**). After PA, no noticeable differences in IL-4 or IL-13 production by microglia in the PFC were observed in the IT group compared to the NIT group (**Figure 3Biv**).

For myeloid cell subsets, there were similar frequency of monocytes and eosinophils before (**Supplementary Figure S3Ci**) and after PA (**Supplementary Figure S3Cii**) of *S. mansoni* infected animals compared with non-infected controls in HPC and PFC. We also noted comparable frequency of CD11b^+^ cells (**Supplementary Figure S3A, B**) and their production of IL-4 (**Supplementary Figures S3Aiii, Biii**) and IL-13 (**Supplementary Figures S3Aiv, Biv**) in HPC and PFC, respectively, in NINT, INT and IT groups. to summarize this part, our results suggest that while schistosomiasis infection did not significantly impact the CD11b^+^ cell phenotype, it did enhance microglia polarization towards an microglia producing TNFα phenotype, particularly in the hippocampus. Importantly, PA might play a role in mitigating microglia adoption of the microglia producing TNFα phenotype by restoring TNFα production to baseline levels. These findings underscore the potential influence of PA in modulating microglia response in the context of schistosomiasis infection and emphasize the need for further investigation into the underlying mechanisms and implications for disease progression.

### Impact of schistosomiasis and training on astrocytes phenotype

Expanding our examination to include other glial cells, specifically astrocytes-GFAP+ cells, we probed into their activity, antigen presentation capacity, and cytokine production in response to schistosomiasis infection.

GFAP-positive astrocytes are generally associated with functions like forming glial scars, modulating inflammation, and protecting surrounding neural tissue.

**Figure 4:**
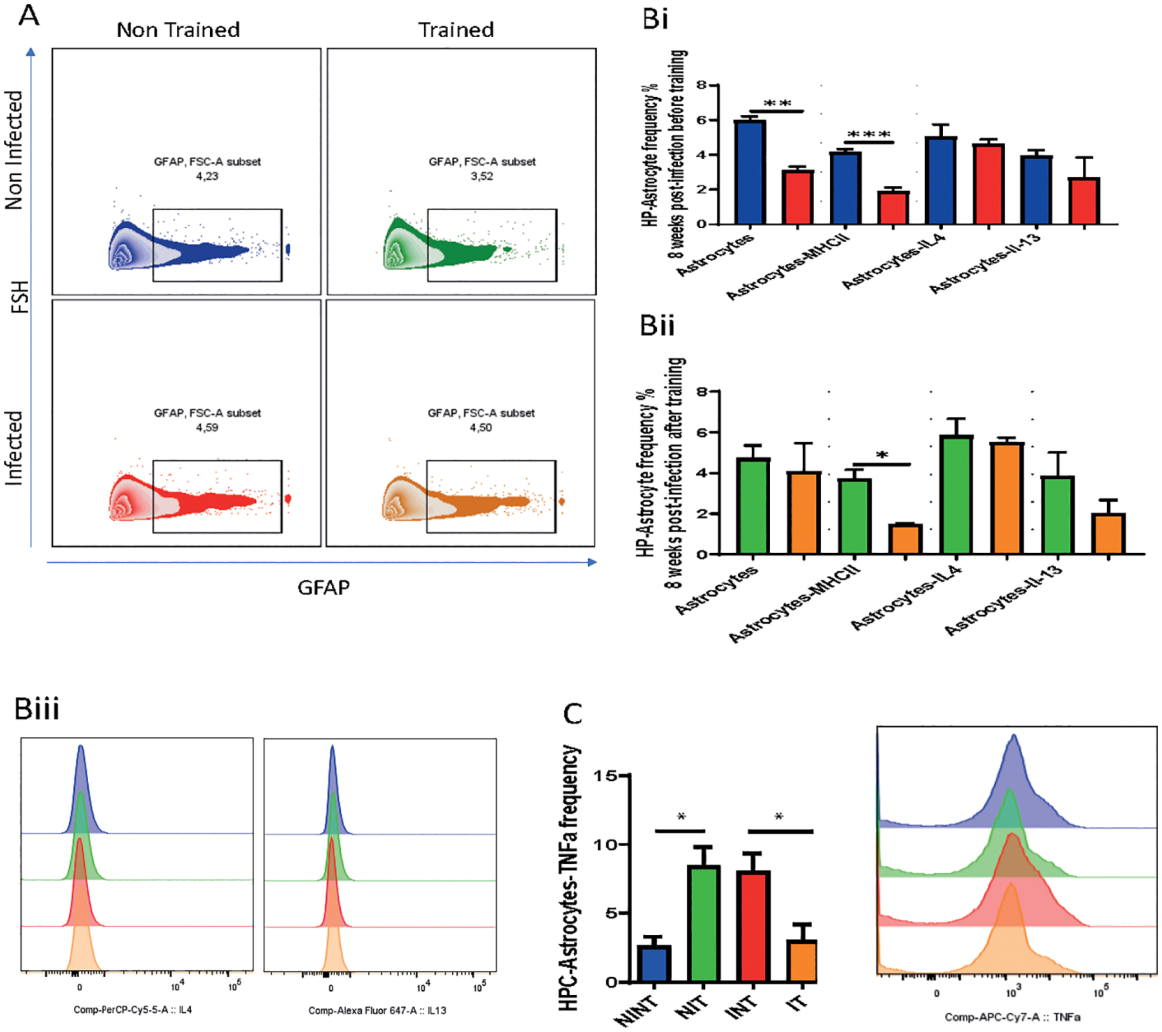
Impact of schistosomiasis infection and/or physical activities on astrocyte phenotype. **(A)** Representative flow cytometry of GFAP^+^ astrocytes in hippocampus. **(Bi)** Frequency of GFAP^+^ astrocytes, MHC class II expression, and astrocytes producing IL-4 and IL-13 before and **(Bii)** after training. **(Biii)** IL-4 (left) and IL-13 (right) expression by astrocytes depicted in **(Bi, Bii)**. **(C)** Frequency of astrocytes producing TNFα assessed using flow cytometry. Results are pooled from two different experiments with 6-8 mice per group. Data are expressed as mean ± S.E.M. NS, P > 0.05; *P < 0.05, **P < 0.001, ***P < 0.0001 by two-way ANOVA followed by Bonferroni.

To understand the changed happening in GFAP+Astrocytes, we measured GFAP expression (**Figure 4A**). Surprisingly, our results revealed a reduction in GFAP expression due to *S. mansoni* infection in the infected non-trained (INT) compared to non-infected non-trained (NINT) group (**Figure 4Bi**). Additionally, PA did not alter astrocyte reactivity, with the IT group showing similar GFAP expression compared to the NIT group (**Figure 4Bii**).

Furthermore, our results indicated that schistosomiasis infection and/or PA led to a significant reduction in astrocyte antigen presentation capacity, marked by lower MHCII expression (**Figures 4Bi, Bii**). The statistical analysis revealed a significant interaction effect, emphasizing the interdependence of infection and PA on astrocyte antigen presentation capacity (**Figures 4Bi, Bii**. However, no significant main effects of PA or infection alone on MHCII expression were observed. These changes may signify a loss of GFAP+ astrocytes that maybe responsible for modulating the inflammation or protecting the neural tissue induced by infection. Importantly, there were no significant alterations in the production of type 2 cytokines, namely IL-4 and IL-13, between the INT and NINT groups (**Figure 4Bi**) or between the NIT and IT groups (**Figure 4Bii**). Of particular note, PA exerted a positive influence on astrocyte production of TNFα compared to the NINT control group. Similarly, schistosomiasis infection induced a significant increase in TNFα production by astrocytes, which returned to baseline levels after training. These compelling findings highlight that, akin to microglia, PA effectively restored TNFα production by astrocytes to baseline levels post-*S. mansoni* infection. We can conclude that, our investigation into GFAP + astrocyte responses underlined the intricate dynamics influenced by schistosomiasis infection and PA. The restoration of TNFα production emphasizes the potential of exercise to modulate astrocyte GFAP+, offering valuable insights into the broader implications for disease progression and therapeutic strategies. In here, It’s important to note that *GFAP* does not capture the full diversity of astrocyte subtypes and states, such as those involved in metabolic support, neurotransmitter regulation, or synaptic modulation. Thus additional study should be conducted.

### Schistosomiasis infection alters meningeal lymphocyte composition

**Figure 5:**
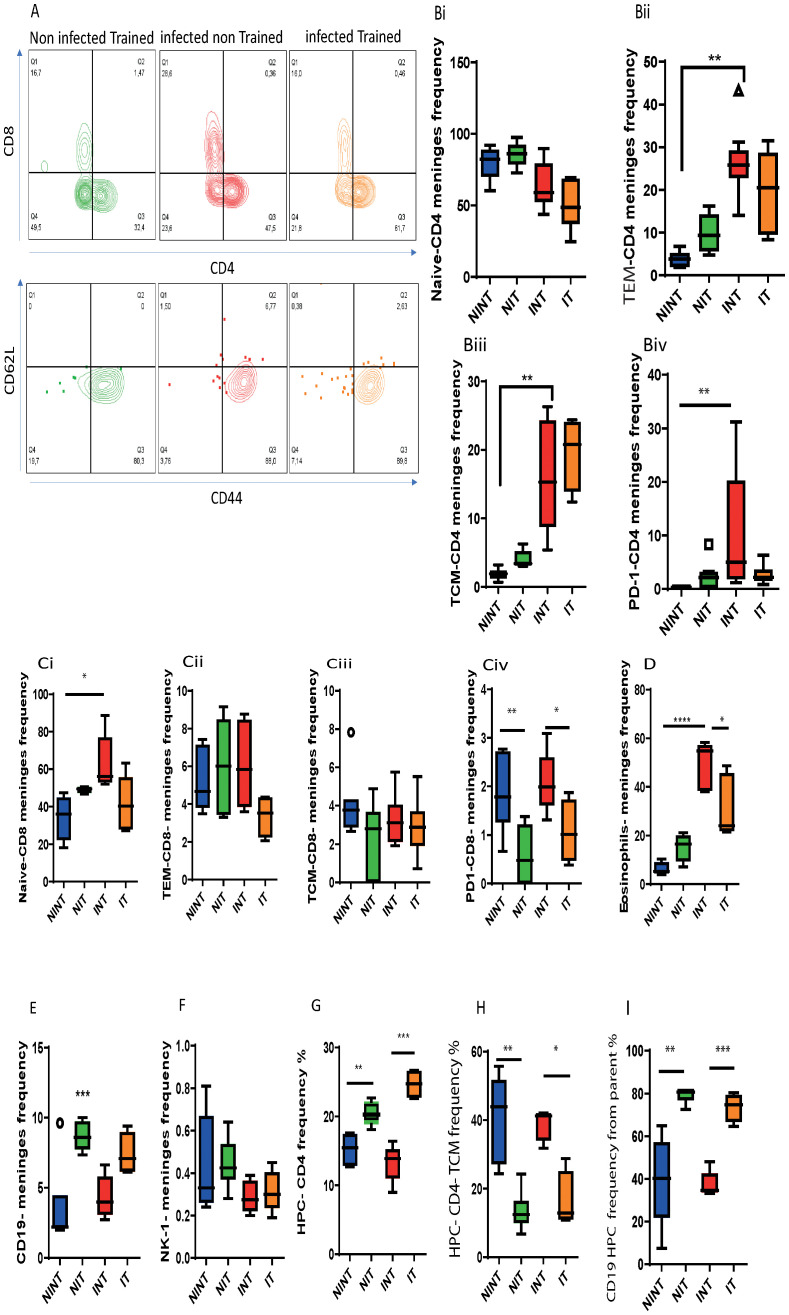
Impact of schistosomiasis infection and/or physical activities on lymphoid and myeloid cells in meninges and brain parenchyma. **(A)** Representative flow cytometry of CD4^+^ and CD8^+^ T cell populations (upper row) and CD62L^+^ CD44^+^ central memory and CD62L^-^ CD44^+^ effector T cell subsets (lower row) in the meninges. **(Bi)** Frequency of naïve, **(Bii)** effector memory (TEM), and **(Biii)** central memory (TCM) subsets and **(Biv)** the expression of PD1 by CD4^+^ T cells. **(Ci)** Frequency of naïve, **(Cii)** effector memory (TEM), and **(Ciii)** central memory (TCM) subsets and **(Civ)** the expression of PD1 by CD8^+^ T cells. Frequency of **(D)** eosinophils, **(E)** CD19^+^ B cells, and NK1.1^+^ cells in the meninges. **(G)** Frequency of total CD4^+^ T cells and **(H)** TCM subset in the HPC. **(I)** Frequency of CD19^+^ B cells in the HPC. Results are pooled from two different experiments with 6-8 mice per group. Data are expressed as mean ± S.E.M. NS, P > 0.05; *P < 0.05, **P < 0.001, ***P < 0.0001, ****P < 0.000. by ANOVA followed by Bonferoni, or unparametric test.

In our pursuit to characterize the influence of schistosomiasis infection on meningeal lymphocyte composition, we employed flow cytometry to phenotype lymphoid and myeloid cell subsets in the meninges of both non-infected and schistosomiasis-infected mice, both before and after PA. Examining T cell subsets (**Figure 5A**), we noted that while a similar frequency of naïve CD4^+^ T cells in the meninges upon *S. mansoni* infection (**Figure 5Bi**), the infection led to a significant increase in the frequency of effector T cells (TEM, **Figure 5Bii**). This effect was influenced by PA,. Additionally, infection alone had a significant effect on both TEM and central memory T cell (TCM) subsets (**Figures 5Bii, Biii**), highlighting an increase in these T cell subsets in the meninges. However, PA alone did not significantly affect the frequency of TEM or TCM CD4^+^ T cell subsets.

Further characterization of the CD4^+^ T cell population in the meninges revealed higher PD1 expression, indicating T cell exhaustion (**Figure 5Biv**). While PA alone did not significantly impact PD1 expression, it effectively countered the schistosomiasis-induced alteration of CD4+ T cell populations. PA reduced the frequency of TEM CD4^+^ T populations without affecting other subpopulations or PD1 expression compared to the infected (INT) group (**Figures 5Bi-Biv**).

Analyzing the CD8^+^ T cell population, schistosomiasis infection led to a significant increase in the naïve CD8^+^ T cell population (**Figure 5Ci**). The impact of training on the frequency of naïve CD8^+^ T cells was influenced by schistosomiasis infection, as indicated by a significant interaction. Notably, PA did not alter the frequency of CD8^+^ T cell subsets (naïve, TEM, and TCM) compared to the training group (**Figures 5Ci-Ciii**). Moreover, PA helped in partially restoring the basal frequency of naïve CD8 (**Figure 5Ci**) and TEM-CD8 (**Figure 5Cii**) subsets, demonstrating its beneficial impact on these subsets in the meninges after infections. Additionally, PA reduced CD8^+^ T cell exhaustion, indicated by decreased PD1 expression (**Figure 5Civ**),.

Further analyses unveiled a significant interaction between PA and infection in the eosinophil population, emphasizing the positive influence of training in mitigating the increase in eosinophils induced by schistosomiasis infection (**Figure 5D**). Additionally, wide-screen analyses demonstrated an increase in various markers (CD62L, CXCR5, SiglecF, CD44, CD45) in response to *S. mansoni* infection, which was reduced through PA in the IT group compared to the INT group (**Supplementary Figure S4**). Furthermore, no significant changes were observed in the CD19^+^ B cell population (**Figure 5E**) or NK1.1^+^ cell population (**Figure 5F**) in the meninges of INT compared to NINT group.

In the brain parenchyma, particularly the HPC, schistosomiasis infection did not induce major changes in lymphoid cell populations. However, PA had a notable impact, enhancing the CD4^+^ T cell (**Figure 5G**) and CD19^+^ B cell populations (**Figure 5I**). Interestingly, the increase in the CD4^+^ T cell population was not driven by the central memory T cell (TCM) population, which was downregulated before and after schistosomiasis infection (**Figure 5H**) but possibly through another T cell subset like TEM. Moreover, an increase in the hippocampus CD 19 ^+^ cell population was observed in the *S. mansoni* infection group after PA (IT) compared to the INT group (**Figure 5I**), this increase is not related to the infection but is related to the benefit impact of PA on hippocampus.

In conclusion, our data illustrate the nuanced effects of schistosomiasis infection and PA on lymphoid and myeloid populations in the meninges and brain parenchyma. PA emerges as a potential mitigator of infection-induced changes in these populations, providing valuable insights into the intricate interplay between PA and immune responses in the context of schistosomiasis.

## Discussion

Schistosomiasis infection correlated with diminished cognitive performance, particularly evident in school-aged children afflicted with heavy or moderate infections, leading to educational, learning, and memory deficits (24, 25). Both the specific cognitive domain affected and the mechanism underlying its pathogenesis is incompletely understood. The current therapeutic approaches carry a lot of side effects, thereby finding a natural alternative is imperative. Whether PA may help in alleviating these consequences is an open question that yet to be explored. In our study, we investigated the effects of schistosomiasis infection on cognitive function in adult mice, delved into the cellular-level alterations in the CNS during the acute stage of infection, and evaluated the potential benefits of PA in mitigating schistosomiasis-induced systemic inflammation on CNS. First, our findings revealed that during the acute infection in adult mice, schistosomiasis infection did not significantly impair simple and complex learning or spatial reference memory, however; it did adversely affect recall memory. At the cellular level, we observed significant alterations in T cell populations and eosinophils within the meninges, accompanied by dysregulated cytokine production in the hippocampus. Additionally, an increase in microglial frequency and activation status, along with heightened TNFα production in response to the infection. Moreover, the infection promoted the expression of muscarinic receptors in the hippocampus, while reducing the number and antigen presentation capacity of astrocytes. Interestingly, PA in infected animals partially mitigated schistosomiasis-induced changes in glial and immune cell phenotypes within the brain parenchyma and meninges, respectively. Most notably, exercise reduced the production of TNFα by glial cells, indicating a shift towards a less pro-inflammatory environment. Collectively, these unprecedented findings shed light on the immune and glial cell alterations within the CNS induced by schistosomiasis and underscore the potential of PA in ameliorating schistosomiasis-induced CNS alterations.

Several studies have indicated that schistosomiasis infection causes significant impairment of brain cognitive functions (3, 25). Whereas schistosomiasis impact on cognitive functions were assessed at the pre-clinical and clinical stages (5, 10), information is very scarce about the specific cognitive domain(s) impacted in adult age. Recent paper on the impact of schistosomiasis in post-natal mice showed that infected animals suffered from impairment in spatial learning and memory formation during acute schistosomiasis (10). By contrast, our findings indicated unaffected simple and complex learning as well as spatial reference memory in adult mice. The infection, however; resulted in impairment in the recall cognitive behavior. Two potential explanations may elucidate these disparities: firstly, schistosomiasis may exert varying impacts on cognitive domains depending on the host’s maturation stage; and secondly, a higher infection inoculum used in the previous study could lead to more severe impacts of schistosomiasis infection on CNS function. Future experiments involving different infection doses administered to mice at various maturation stages will be instrumental in developing a more comprehensive understanding in that regard. Regarding affective behavior, emotions-associated behaviour, schistosomiasis infection did neither alter locomotor activity nor anxiety levels, as infected mice exhibited similar distances moved and velocities compared to uninfected controls. These findings are consistent with a previous report showing that infection of postnatal mice did neither alter locomotion nor anxiety level (10), and in contrast with others that demonstrated impact of helminth infection on the locomotion, suggesting specific species-neurobehavior impact (26, 27). Together, these findings suggested that while schistosomiasis infection had a specific effect on spatial reference memory, it had minimal influence on motor performance and affective behavior in adult mice.

Due to the spine and the large size of *S. mansoni* eggs, they are almost unable to traverse the blood brain barrier and they usually infiltrate the lower domain of the spinal cord (5, 28, 29). In agreement, our histological examination of brain sections indicated absence of eggs translocation into the brain. We also noted absence of egg infestation in the spinal cord likely due to the acute stage of the disease, which is one of the limitation in this study. Therefore, the noted schistosomiasis impact on the CNS was most likely through the systemic inflammation. This systemic inflammation led to significant alteration in T cell profile in meninges. In fact, there was a remarkable increase in effector and central memory CD4^+^ T cell population that was associated with increased cell exhaustion as indicated by higher level of PD-1 expression. We also noted an increase in naïve CD8^+^ T cell population. These alterations in T cell population in the brain could be the causal of significant impairment of hippocampal-dependent spatial learning and memory acquisition (30, 31). Hence, the changes in T cell population could be driving the schistosomiasis-induced impairment of the recall cognitive functions.

Schistosomiasis infection led also to a significant increase in the frequency of eosinophil population in the meninges, the phenomenon previously demonstrated in *T. regenti* infection (26). Further investigation is warranted to determine whether the elevated infiltration of eosinophils was driven by increased production of IL-5 by T cells (32).

By affecting the cell dynamics in the meninges, neuroinflammation in the brain parenchyma was highly anticipated. In agreement, there was an increase in microglia frequency and activation in hippocampus, while maintaining similar frequency and reactivity in prefrontal cortex, suggesting a profound impact of schistosomiasis infection on HPC compared to prefrontal cortex at adult stage. Significant increase in microglia was also recorded in the CNS of schistosomatidae infected mice (26). Investigating microglia polarization indicated a significant increase in TNFα production in the infected group compared to non-infected non-trained control. While TNFα production can promote inflammation and edema in addition to its toxic effects on neuronal structure and myelin, it could also promote neural cell survival and proliferation (13). The tendency of one effect over another is context dependent. In the present study, the predominant production of TNFα over type 2 cytokines (IL-4 and IL-13) could suggest the domination of pro-inflammatory effects of TNFα during neuroschistosomiasis after 8 weeks post-infections. Microglia production of TNFα may initiated neuroinflammation and aggravate neural tissue injury. It could further impair the neurogenesis and synaptogenesis in hippocampus (33) which may explain the dysregulation in recall memory (34, 35). The predomination of pro-inflammatory environment in hippocampus during neuroschistosomiasis could be the cause of the increased cell infiltration and reduction in the area occupied [the quantification of cell presence within a defined spatial area, specifically calculated as the number of cells per cubic millimeter (mm³)] in the prefrontal cortex but not the hippocampus.

Cytokine profile in brain parenchyma was also significantly altered. We noted a consistent increase in IL-6 in hippocampus and prefrontal cortex, and IL-4 and TNFα in HPC. The higher level of IL-6 and IL-4 is consistent with the previous study demonstrated an increase in the same cytokines in cerebrospinal fluid (CSF) in spinal cord schistosomiasis (SCS) patients (36). Schistosomiasis infection at oviposition stage, > 6 weeks post-infection, is characterized by the domination of type 2 immunity (4). Hence, the production of IL-4 and IL-6 could be helping in skewing the immune response to type 2 immunity in the CNS to downmodulate pro-inflammatory immune response and diminish reactive oxygen and nitrogen species production that may damage the tissue. In fact, they could possibly mitigate the increased expression level of TNFα in hippocampus produced by microglia and astrocytes in response to the infection. The increase in TNFα is in disagreement with previous attempts by other investigator showing lower TNFα in CSF of SCS patients (36) and similar TNFα in prefrontal cortex in schistosomiasis-infected mice at postnatal stage (10). Of note, IL-6 may also induce nervous tissue lesion. Future studies will be needed to dissect the independent functional contributions of these cytokine to schistosomiasis effects on CNS.

Schistosomiasis infection further induced alteration in the cholinergic system. This could be due to the reliance of schistosomiasis parasites on the neurotransmitters produced by the host for the activation of their nervous system (37). Schistosomiasis nervous system is principal in the parasites survival, motility, nutrient uptake, and reproduction. ACh is one of the most important neurotransmitters needed for muscles contraction and nutrient absorption (38–40). Enhanced ACh expression by the cells in the spinal cord could then be driven by the parasites in favor of their survival. The increased production of ACh was paralleled by enhanced expression of the muscarinic receptors in the hippocampus namely, M1, M2 and M5. Recently, it was shown that systemic schistosomiasis infection results in enhanced phosphorylation of Tau, microtubule associated protein (10). Increased uptake of Tau by neurons through M1 and M3 receptors enhanced microglia activation while maintaining similar GFAP expression level (41). Our results partially goes in line with these findings as we noted increase in microglia population and similar GFAP expression. However, the frequency of GFAP^+^ astrocytes was significantly diminished in infected animals and were characterized by their potency of producing higher level of TNFα. The changes in muscarinic receptors expression could be playing a role in the noted immune dysregulation (42).

The utilization of physical activity (PA) as an adjunctive therapy in the treatment of neuroschistosomiasis represents an innovative approach to modulate host immune responses and potentially ameliorate disease outcomes. Praziquantel (PZQ) remains the primary antihelmintic drug for treating schistosomiasis, and its efficacy is well-established; it can reduce neuroschistosomiasis symptoms as school-aged children treated with PZQ often show enhanced cognitive performance, including improvements in attention, memory, and problem-solving skills. However, while PZQ effectively kills the parasites, it does not directly address the inflammatory symptoms and complications associated with neuroschistosomiasis. Consequently, adjunctive therapies like corticosteroids are frequently needed to manage symptoms, which comes with their own set of potential side effects (5, 6). Previous studies have found a protective effect of PA against inflammation-associated diseases (11, 12).

Therefore, in our study, we explored the intricate effects of an 8-day PA regimen on glial and immune cells within the CNS of schistosomiasis-infected subjects. Our data revealed that PA not only mitigated neuroinflammation but also fostered a milieu conducive to neuroprotection. The dampening of TNFα production and the concurrent increase in IL-13 within the hippocampus suggested a potential shift toward an anti-inflammatory state. This was particularly pertinent given the neurotoxic potential of chronic TNFα elevation and the neuroprotective role IL-13 can play. Exercise-induced modulation of glial cell activity, characterized by the normalization of pro-inflammatory cytokine expression, indicated that PA exerted a regulatory influence over CNS innate immunity, which could have broad implications for limiting the neurological impact of schistosomiasis.

Furthermore, PA had a restorative effect on neurotransmitter system markers, such as ACh in the spinal cord and M5 muscarinic receptors in the hippocampus, which may indicate a broader impact on CNS function and disease resilience. The PA regimen positively influenced lymphocyte populations, reducing the frequency of CD4+ and CD8+ effector memory T cells and eosinophils within the meninges. These could further alleviate meningeal inflammation and reduce the risk of long-term structural CNS damage. These neuro-immunological adjustments underscored the capacity of PA to act beyond a simple physical enhancer and position it as a powerful modulator of central immune and neural processes.

However, despite these beneficial CNS effects, PA unexpectedly influenced other disease dynamics. We previously demonstrated that gut smooth muscles hypercontractility is crucial for egg expulsion and any defects in this process led to higher susceptibility to the infection and premature death (23). Consequently, we expected that enhancing gut smooth muscle hypercontractility by PA would help in reducing egg burden but in contrary we noted significant increase in the egg burden after training. This unexpected finding suggested that PA could somehow have interfered with the egg expulsion process, possibly by redirecting the body’s activity in another direction. We also noted worsened the egg-induced granuloma and fibrosis. One possible explanation could be that increase in IL-6 and C-reactive protein usually generated during PA could have led to the exacerbation of the disease immunopathology (11). Given these findings, it is crucial to understand the balance between beneficial and potentially detrimental effects of PA. Our results thus necessitate a careful consideration of the role of exercise in the management of schistosomiasis, highlighting the importance of future research to delineate the optimal types and intensities of PA that could improve disease outcomes without worsening the peripheral pathology, particularly concerning liver health and fibrosis, which are not ameliorated by PA according to previous studies.

Overall, our results demonstrated the impact of schistosomiasis infection on the cognitive function and cellular composition and phenotype in the CNS and how regular moderate PA could help in mitigating the impact of schistosomiasis on the CNS. Our results indicated impairment specifically in the recall memory during acute stage of the infection in adult mice. We further demonstrated the changes in glial and immune cells in CNS. Our results illustrated changes in T cells and eosinophils in the meninges which were accompanied by higher the propensity of glial cell to produce pro-inflammatory cytokines, particularly TNFα during schistosomiasis infection. We then highlighted how PA alleviated the schistosomiasis-induced impacts on the CNS. Together, our data support that moderate regular PA could be a natural non-invasive strategy that could help in mitigating the impact of *S. mansoni* infection on CNS.

## Data Availability

The datasets presented in this study can be found in online repositories. The names of the repository/repositories and accession number(s) can be found in the article/**Supplementary Material**.

## Supplementary material

Supplementary Figure 1 Impact of physical activities on the periphery response to schistosomiasis infection. 8 weeks p.i. 1 cm jejunum segment was removed from the small intestine of both non-infected and *S. mansoni* infected mice to assess the smooth muscle hypercontractility. **(A)** Smooth muscle hypercontractility measured using acetylcholine (ACh). jejunum segment was stimulated with varying concentrations of ACh −9 to −3 LOG [M] to determine isometric contractile responses. **(B)** Egg burden in liver, **(C)** intestine, **(D)** spleen, and **(E)** lung. **(F)** Representative H&E staining of liver sections from mice infected with *S. mansoni* for 8 weeks before (INT) and after training (IT) (4x). **(G)** Liver Granuloma size determined from **(F)** by using a computerized morphometric analysis program (NIS elements by NIKON) by measuring 100 granulomas/group. **(H)** Liver hydroxyproline content measured by colorimetry 8 weeks p.i. **(I)** Schema of second postinfection Training (11weeks PI) **(Ii)** Latency to platform after 11 weeks postinfection, **(Iii)**, latency of platform in day 6,7,8, after 11 days postinfection, **(Iiii)** Percentage time in quadrant 11 weeks postinfections. Results are pooled from two different experiments with 6-8 mice per group. Data are expressed as mean ± S.E.M. NS, P > 0.05; * *P* < 0.05, ** *P* < 0.001, *** *P* < 0.0001 by two-tailed unpaired *Student t* test,.

Supplementary Figure 2 Impact of schistosomiasis infection and/or physical activities on spinal cord. **(A)** Representative H&E sections for spinal cord to assess eggs infiltration (original magnification 4 and10x). **(B)** Egg burden in spinal cord. **(Ci)**) cholinergic neurons in coronal sections of spinal cord For astrocytes producing acetylcholine in the spinal cord (original magnification 10x). **(Cii)** Number of cell producing ACh in the spinal cord. **(Di)** *Ccl2*, **(Dii)** *Il13*, and **(Diii)** *Il4* relative to HPRT housekeeping gene determined using qRT-PCR in prefrontal cortex, mRNA expression level of **(Div)** *M1*, **(Dv)** *M2*, and **(Dvi)** *M5* relative to HPRT housekeeping gene determined using qRT-PCR in prefrontal cortex Results are pooled from two different experiments with 6-8 mice per group. Data are expressed as mean ± S.E.M. NS, P > 0.05; * *P* < 0.05, ** *P* < 0.001, *** *P* < 0.0001 by two-tailed by ANOVA followed by Bonferoni,.

Supplementary Figure 3 Impact of schistosomiasis infection and/or physical activities on CD11b^+^ cells in brain parenchyma. **(A)** Frequency of CD11b^+^ cells in HPC 8 weeks p.i. **(Ai)** IL-4 and **(Aii)** IL-13 expression by CD11b^+^ cells in hippocampus. **(B)** Frequency of CD11b^+^ cells in PFC 8 weeks p.i. **(Bi)** IL-4 and **(Bii)** IL-13 expression by CD11b^+^ cells in prefrontal cortex. **(Ci)** Frequency of monocytes and eosinophils in hippocampus and prefrontal cortex before and **(Cii)** after mice training. **(D)** Parenchyma gating strategy, **(E)** Meningeal gating strategy. Results are pooled from two different experiments with 6-8 mice per group. Data are expressed as mean ± S.E.M. NS, P > 0.05; * *P* < 0.05, ** *P* < 0.001, *** *P* < 0.0001 by two-tailed by ANOVA followed by Bonferoni, or unparametric test.

Supplementary Figure 4 UMAP for the Impact of schistosomiasis infection and/or physical activities on immune cells in brain, Wide screen analyses of CD62L, CXCR5, SiglecF, CD3, CD4, NK1.1, CD45, PD-1, CD8 demonstrated a remarkable increase in CXCR5, SiglecF, CD44, CD45 expression in the meninges in response to *S. mansoni* infection.

## References

[1] Steinmann P Keiser J Bos R Tanner M Utzinger J. Schistosomiasis and water resources development: systematic review, meta-analysis, and estimates of people at risk. Lancet Infect Dis. (2006) 6:411–25. doi: 10.1016/S1473-3099(06)70521-7 16790382

[2] Utzinger J Raso G Brooker S De Savigny D Tanner M Ornbjerg N. Schistosomiasis and neglected tropical diseases: towards integrated and sustainable control and a word of caution. Parasitology. (2009) 136:1859–74. doi: 10.1017/S0031182009991600 PMC2791839 19906318

[3] McManus DP Dunne DW Sacko M Utzinger J Vennervald BJ Zhou XN. Schistosomiasis. Nat Rev Dis Primers. (2018) 4:13. doi: 10.1038/s41572-018-0013-8 30093684

[4] Abdel Aziz N Musaigwa F Mosala P Berkiks I Brombacher F. Type 2 immunity: a two-edged sword in schistosomiasis immunopathology. Trends Immunol. (2022) 43:657–73. doi: 10.1016/j.it.2022.06.005 35835714

[5] Ferrari TC Moreira PR. Neuroschistosomiasis: clinical symptoms and pathogenesis. Lancet Neurol. (2011) 10:853–64. doi: 10.1016/S1474-4422(11)70170-3 21849166

[6] Ross AG McManus DP Farrar J Hunstman RJ Gray DJ Li YS. Neuroschistosomiasis. J Neurol. (2012) 259:22–32. doi: 10.1007/s00415-011-6133-7 21674195

[7] Carod Artal FJ Vargas AP Horan TA Marinho PB Coelho Costa PH. *Schistosoma mansoni* myelopathy: clinical and pathologic findings. Neurology. (2004) 63:388–91. doi: 10.1212/01.WNL.0000130190.67613.BE 15277648

[8] Caumes E Vidailhet M. Acute neuroschistosomiasis: a cerebral vasculitis to treat with corticosteroids not praziquantel. J Travel Med. (2010) 17:359; author reply 60. doi: 10.1111/j.1708-8305.2010.00452_1.x 20920061

[9] Jaureguiberry S Paris L Caumes E. Acute schistosomiasis, a diagnostic and therapeutic challenge. Clin Microbiol Infect. (2010) 16:225–31. doi: 10.1111/j.1469-0691.2009.03131.x 20222897

[10] Gasparotto J Senger MR Telles de Sa Moreira E Brum PO Carazza Kessler FG Peixoto DO. Neurological impairment caused by *Schistosoma mansoni* systemic infection exhibits early features of idiopathic neurodegenerative disease. J Biol Chem. (2021) 297:100979. doi: 10.1016/j.jbc.2021.100979 34303703 PMC8361297

[11] Gleeson M Bishop NC Stensel DJ Lindley MR Mastana SS Nimmo MA. The anti-inflammatory effects of exercise: mechanisms and implications for the prevention and treatment of disease. Nat Rev Immunol. (2011) 11:607–15. doi: 10.1038/nri3041 21818123

[12] Khazaei M. Chronic low-grade inflammation after exercise: controversies. Iran J Basic Med Sci. (2012) 15:1008–9. PMC3586919 23495361

[13] Hanisch UK. Microglia as a source and target of cytokines. Glia. (2002) 40:140–55. doi: 10.1002/glia.10161 12379902

[14] Vitkovic L Maeda S Sternberg E. Anti-inflammatory cytokines: expression and action in the brain. Neuroimmunomodulation. (2001) 9:295–312. doi: 10.1159/000059387 12045357

[15] Vorhees CV Williams MT. Morris water maze: procedures for assessing spatial and related forms of learning and memory. Nat Protoc. (2006) 1:848–58. doi: 10.1038/nprot.2006.116 PMC2895266 17406317

[16] Cheever AW. Relative resistance of the eggs of human schistosomes to digestion in potassium hydroxide. Bull World Health Organization. (1970) 43:601–3. PMC2427772 5313073

[17] Nono JK Ndlovu H Abdel Aziz N Mpotje T Hlaka L Brombacher F. Host regulation of liver fibroproliferative pathology during experimental schistosomiasis via interleukin-4 receptor alpha. PloS Negl Trop Dis. (2017) 11:e0005861. doi: 10.1371/journal.pntd.0005861 28827803 PMC5578697

[18] Abdel Aziz N Nono JK Mpotje T Brombacher F. The Foxp3+ regulatory T-cell population requires IL-4Ralpha signaling to control inflammation during helminth infections. PloS Biol. (2018) 16:e2005850. 30379806 10.1371/journal.pbio.2005850 PMC6231676

[19] Abdel Aziz N Berkiks I Mosala P Brombacher TM Brombacher F. Environmental and microbial factors influence affective and cognitive behavior in C57BL/6 sub-strains. Front Immunol. (2023) 14:1139913. doi: 10.3389/fimmu.2023.1139913 37180163 PMC10166845

[20] Pasciuto E Burton OT Roca CP Lagou V Rajan WD Theys T. Microglia require CD4 T cells to complete the fetal-to-adult transition. Cell. (2020) 182:625–40 e24. doi: 10.1016/j.cell.2020.06.026 32702313 PMC7427333

[21] McInnes L Healy J Melville J. UMAP: uniform manifold approximation and projection for dimension reduction. arXiv. (2018). doi: 10.21105/joss.00861

[22] Tamgue O Gcanga L Ozturk M Whitehead L Pillay S Jacobs R. Differential Targeting of c-Maf, Bach-1, and Elmo-1 by microRNA-143 and microRNA-365 Promotes the Intracellular Growth of Mycobacterium tuberculosis in Alternatively IL-4/IL-13 Activated Macrophages. Front Immunol. (2019) 10:421. doi: 10.3389/fimmu.2019.00421 30941122 PMC6433885

[23] Marillier RG Brombacher TM Dewals B Leeto M Barkhuizen M Govender D. IL-4Ralpha-responsive smooth muscle cells increase intestinal hypercontractility and contribute to resistance during acute Schistosomiasis. Am J Physiol Gastrointest Liver Physiol. (2010) 298:G943–51. doi: 10.1152/ajpgi.00321.2009 20360135

[24] Jukes MC Nokes CA Alcock KJ Lambo JK Kihamia C Ngorosho N. Heavy schistosomiasis associated with poor short-term memory and slower reaction times in Tanzanian schoolchildren. Trop Med Int Health. (2002) 7:104–17. 10.1046/j.1365-3156.2002.00843.x 11841700

[25] Ezeamama AE Bustinduy AL Nkwata AK Martinez L Pabalan N Boivin MJ. Cognitive deficits and educational loss in children with schistosome infection-A systematic review and meta-analysis. PloS Negl Trop Dis. (2018) 12:e0005524. doi: 10.1371/journal.pntd.0005524 29329293 PMC5766129

[26] Machacek T Leontovyc R Smidova B Majer M Vondracek O Vojtechova I. Mechanisms of the host immune response and helminth-induced pathology during Trichobilharzia regenti (Schistosomatidae) neuroinvasion in mice. PloS Pathog. (2022) 18:e1010302. 35120185 10.1371/journal.ppat.1010302 PMC8849443

[27] Janecek E Waindok P Bankstahl M Strube C. Abnormal neurobehaviour and impaired memory function as a consequence of Toxocara canis- as well as Toxocara cati-induced neurotoxocarosis. PloS Negl Trop Dis. (2017) 11:e0005594. doi: 10.1371/journal.pntd.0005594 28481889 PMC5436879

[28] Carod-Artal FJ. Neurological complications of Schistosoma infection. Trans R Soc Trop Med Hyg. (2008) 102:107–16. doi: 10.1016/j.trstmh.2007.08.004 17905371

[29] Cervellati C Trentini A Pecorelli A Valacchi G. Inflammation in neurological disorders: the thin boundary between brain and periphery. Antioxid Redox Signal. (2020) 33:191–210. doi: 10.1089/ars.2020.8076 32143546

[30] Derecki NC Cardani AN Yang CH Quinnies KM Crihfield A Lynch KR. Regulation of learning and memory by meningeal immunity: a key role for IL-4. J Exp Med. (2010) 207:1067–80. doi: 10.1084/jem.20091419 PMC2867291 20439540

[31] Serre-Miranda C Roque S Santos NC Portugal-Nunes C Costa P Palha JA. Effector memory CD4(+) T cells are associated with cognitive performance in a senior population. Neurol Neuroimmunol Neuroinflamm. (2015) 2:e54. doi: 10.1212/NXI.0000000000000054 25566544 PMC4277304

[32] Kouro T Takatsu K. IL-5- and eosinophil-mediated inflammation: from discovery to therapy. Int Immunol. (2009) 21:1303–9. doi: 10.1093/intimm/dxp102 19819937

[33] Butovsky O Ziv Y Schwartz A Landa G Talpalar AE Pluchino S. Microglia activated by IL-4 or IFN-gamma differentially induce neurogenesis and oligodendrogenesis from adult stem/progenitor cells. Mol Cell Neurosci. (2006) 31:149–60. doi: 10.1016/j.mcn.2005.10.006 16297637

[34] Monje ML Toda H Palmer TD. Inflammatory blockade restores adult hippocampal neurogenesis. Science. (2003) 302:1760–5. doi: 10.1126/science.1088417 14615545

[35] Steadman PE Xia F Ahmed M Mocle AJ Penning ARA Geraghty AC. Disruption of oligodendrogenesis impairs memory consolidation in adult mice. Neuron. (2020) 105:150–64 e6. doi: 10.1016/j.neuron.2019.10.013 31753579 PMC7579726

[36] Ferrari TC Moreira PR Sampaio MJ da Cunha AS de Oliveira JT Gazzinelli G. Intrathecal cytokines in spinal cord schistosomiasis. J Neuroimmunol. (2006) 177:136–41. doi: 10.1016/j.jneuroim.2006.05.008 16822551

[37] Ribeiro P El-Shehabi F Patocka N. Classical transmitters and their receptors in flatworms. Parasitology. (2005) 131 Suppl:S19–40. doi: 10.1017/S0031182005008565 16569290

[38] Camacho M Agnew A. Schistosoma: rate of glucose import is altered by acetylcholine interaction with tegumental acetylcholine receptors and acetylcholinesterase. Exp Parasitol. (1995) 81:584–91. doi: 10.1006/expr.1995.1152 8543000

[39] Jones AK Bentley GN Oliveros Parra WG Agnew A. Molecular characterization of an acetylcholinesterase implicated in the regulation of glucose scavenging by the parasite Schistosoma. FASEB J. (2002) 16:441–3. doi: 10.1096/fj.01-0683fje 11821256

[40] You H Liu C Du X McManus DP. Acetylcholinesterase and nicotinic acetylcholine receptors in schistosomes and other parasitic helminths. Molecules. (2017) 22. doi: 10.3390/molecules22091550 PMC6151654 28906438

[41] Morozova V Cohen LS Makki AE Shur A Pilar G El Idrissi A. Normal and pathological tau uptake mediated by M1/M3 muscarinic receptors promotes opposite neuronal changes. Front Cell Neurosci. (2019) 13:403. doi: 10.3389/fncel.2019.00403 31555098 PMC6737038

[42] Kudlak M Tadi P. Physiology, Muscarinic Receptor. Treasure Island (FL: StatPearls (2022). 32310369

