# Supplementary figures and images for: Moderate regular physical exercise can help in alleviating the systemic impact of schistosomiasis infection on brain cognitive function
Source: Front Immunol. 2025 Jan 31;15:1453742. doi: 10.3389/fimmu.2024.1453742 (PMC11825816; doi:10.3389/fimmu.2024.1453742)

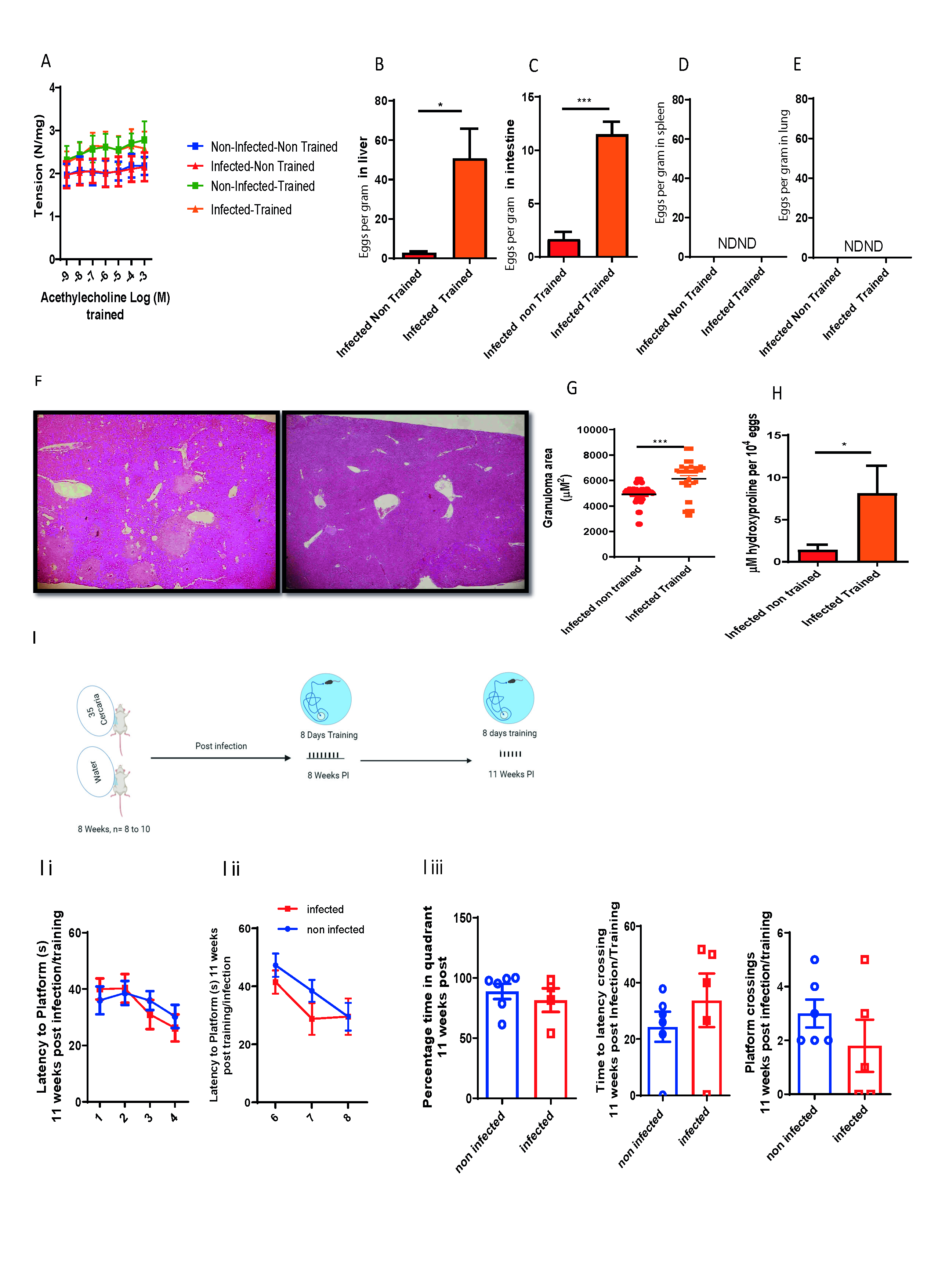

Supplement: Supplementary Figure 1 — Impact of physical activities on the periphery response to schistosomiasis infection. 8 weeks p.i. 1 cm jejunum segment was removed from the small intestine of both non-infected and S. mansoni infected mice to assess the smooth muscle hypercontractility. (A) Smooth muscle hypercontractility measured using acetylcholine (ACh). jejunum segment was stimulated with varying concentrations of ACh −9 to −3 LOG [M] to determine isometric contractile responses. (B) Egg burden in liver, (C) intestine, (D) spleen, and (E) lung. (F) Representative H&E staining of liver sections from mice infected with S. mansoni for 8 weeks before (INT) and after training (IT) (4x). (G) Liver Granuloma size determined from (F) by using a computerized morphometric analysis program (NIS elements by NIKON) by measuring 100 granulomas/group. (H) Liver hydroxyproline content measured by colorimetry 8 weeks p.i. (I) Schema of second postinfection Training (11weeks PI) (Ii) Latency to platform after 11 weeks postinfection, (Iii), latency of platform in day 6,7,8, after 11 days postinfection, (Iiii) Percentage time in quadrant 11 weeks postinfections. Results are pooled from two different experiments with 6-8 mice per group. Data are expressed as mean ± S.E.M. NS, P > 0.05; * P < 0.05, ** P < 0.001, *** P < 0.0001 by two-tailed unpaired Student t test,. [file Image1.tif]

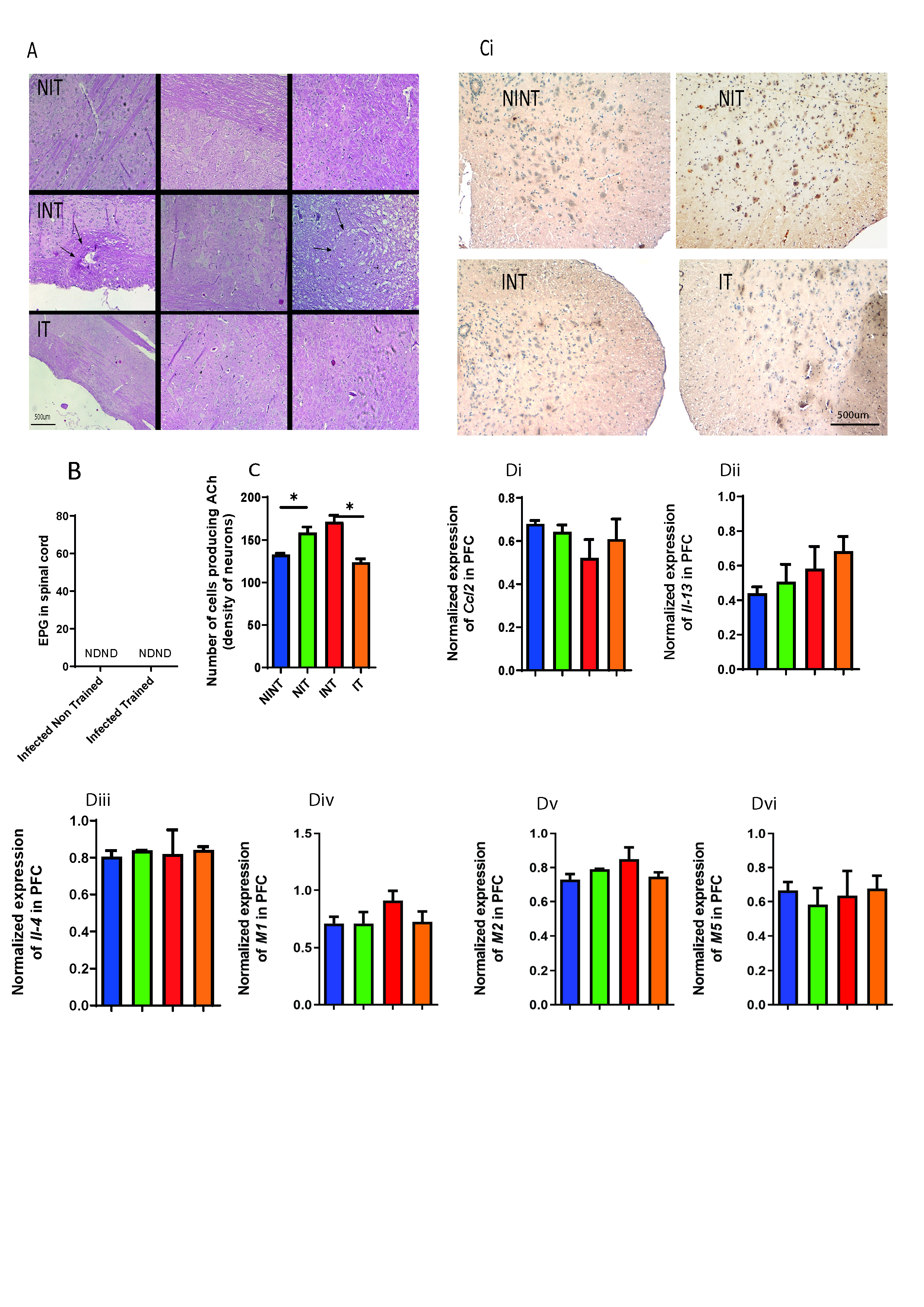

Supplement: Supplementary Figure 2 — Impact of schistosomiasis infection and/or physical activities on spinal cord. (A) Representative H&E sections for spinal cord to assess eggs infiltration (original magnification 4 and10x). (B) Egg burden in spinal cord. (Ci)) cholinergic neurons in coronal sections of spinal cord For astrocytes producing acetylcholine in the spinal cord (original magnification 10x). (Cii) Number of cell producing ACh in the spinal cord. (Di) Ccl2, (Dii) Il13, and (Diii) Il4 relative to HPRT housekeeping gene determined using qRT-PCR in prefrontal cortex, mRNA expression level of (Div) M1, (Dv) M2, and (Dvi) M5 relative to HPRT housekeeping gene determined using qRT-PCR in prefrontal cortex Results are pooled from two different experiments with 6-8 mice per group. Data are expressed as mean ± S.E.M. NS, P > 0.05; * P < 0.05, ** P < 0.001, *** P < 0.0001 by two-tailed by ANOVA followed by Bonferoni,. [file Image2.tif]

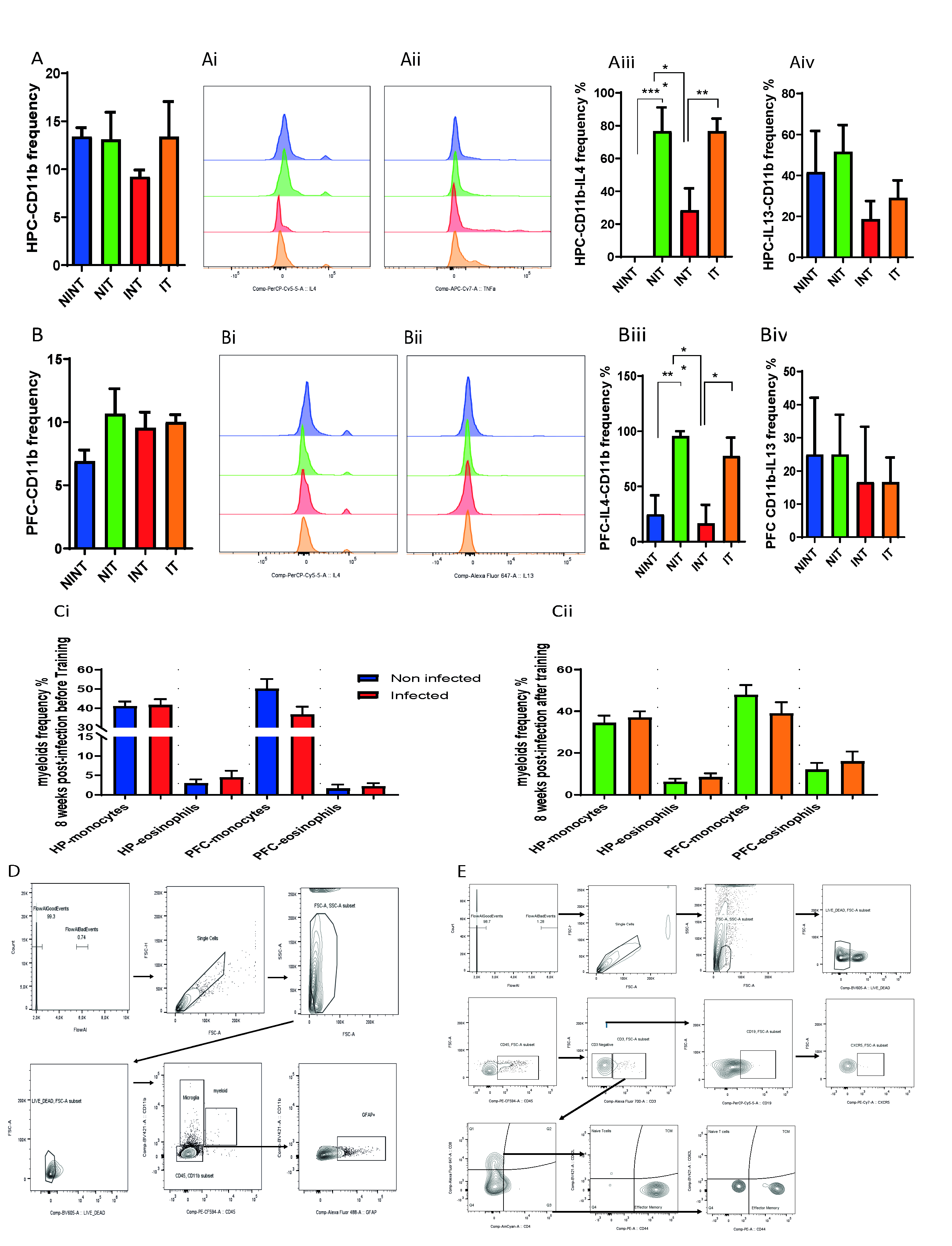

Supplement: Supplementary Figure 3 — Impact of schistosomiasis infection and/or physical activities on CD11b+ cells in brain parenchyma. (A) Frequency of CD11b+ cells in HPC 8 weeks p.i. (Ai) IL-4 and (Aii) IL-13 expression by CD11b+ cells in hippocampus. (B) Frequency of CD11b+ cells in PFC 8 weeks p.i. (Bi) IL-4 and (Bii) IL-13 expression by CD11b+ cells in prefrontal cortex. (Ci) Frequency of monocytes and eosinophils in hippocampus and prefrontal cortex before and (Cii) after mice training. (D) Parenchyma gating strategy, (E) Meningeal gating strategy. Results are pooled from two different experiments with 6-8 mice per group. Data are expressed as mean ± S.E.M. NS, P > 0.05; * P < 0.05, ** P < 0.001, *** P < 0.0001 by two-tailed by ANOVA followed by Bonferoni, or unparametric test. [file Image3.tif]

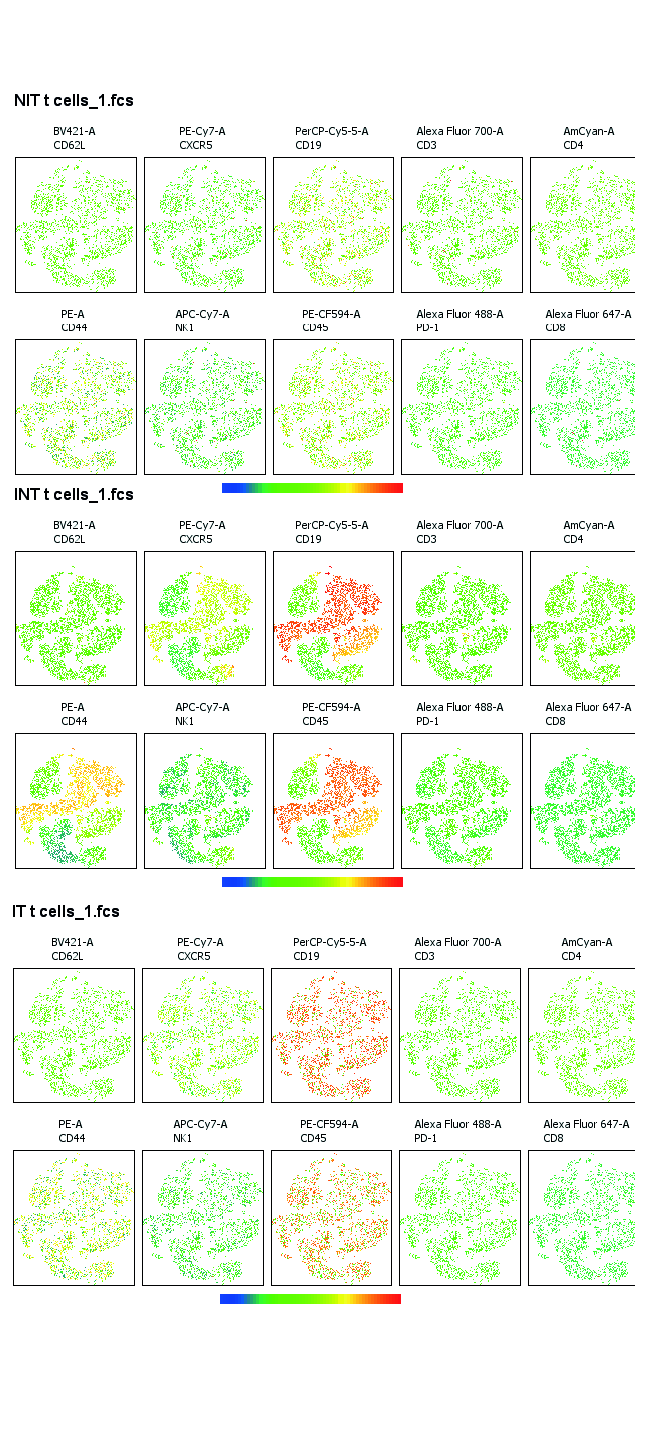

Supplement: Supplementary Figure 4 — UMAP for the Impact of schistosomiasis infection and/or physical activities on immune cells in brain, Wide screen analyses of CD62L, CXCR5, SiglecF, CD3, CD4, NK1.1, CD45, PD-1, CD8 demonstrated a remarkable increase in CXCR5, SiglecF, CD44, CD45 expression in the meninges in response to S. mansoni infection. [file Image4.tif]

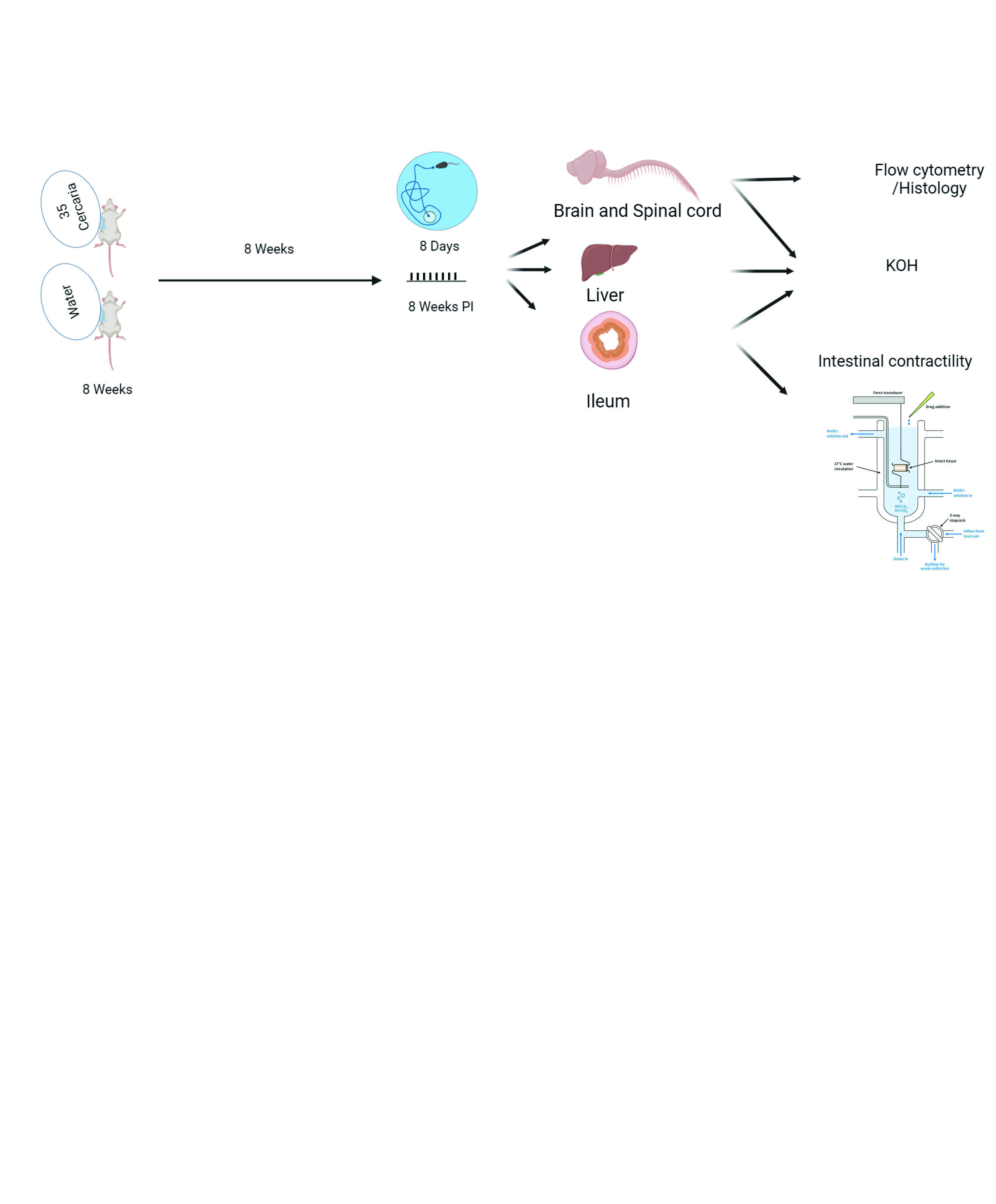

Supplement: Supplementary file 5 [file Image5.tiff]
